# Discovery of new bianthrones and chlorinated bianthrones with cytotoxic activity against cancer cells from *Penicillium hispanicum* guided by HSQC-based DeepSAT

**DOI:** 10.1080/21501203.2025.2526766

**Published:** 2025-07-10

**Authors:** Ruiyun Huo, Minhui Ji, Gaoran Liu, Ling Liu

**Affiliations:** a State Key Laboratory of Microbial Diversity and Innovative Utilization, Institute of Microbiology, Chinese Academy of Sciences, Beijing, China; b College of Life Sciences, University of Chinese Academy of Sciences, Beijing, China

**Keywords:** Mangrove-derived fungus, *Penicillium hispanicum*, HSQC-based DeepSAT, cytotoxic activity, network pharmacology

## Abstract

Three new racemic bianthrones, including two pairs of (±)-penithrones A (**1**) and B (**2**), and a chlorinated derivative (±)-penithrone C (**3**), along with their biogenetic precursors (**4**–**6**) were discovered from the culture extract of the mangrove-derived fungus *Penicillium hispanicum* LA032 using HSQC-based DeepSAT. The structural elucidation of these new compounds was achieved through comprehensive integration of NMR spectroscopy, high-resolution mass spectrometry (HRESIMS), and NMR calculations with CP3 analysis. Compounds **1** and **2** exhibited significant cytotoxic activity against HeLa, HCT116, and MCF-7 cancer cell lines, with IC_50_ values ranging from 5.09 ± 0.65 to 9.47 ± 0.22 μmol/L. In addition, network pharmacology analysis and molecular docking studies revealed Mitogen-Activated Protein Kinase 10 (MAPK10) as a potential target of **1** for its anticancer effect.

## GRAPHICAL ABSTRACT

**Figure:**
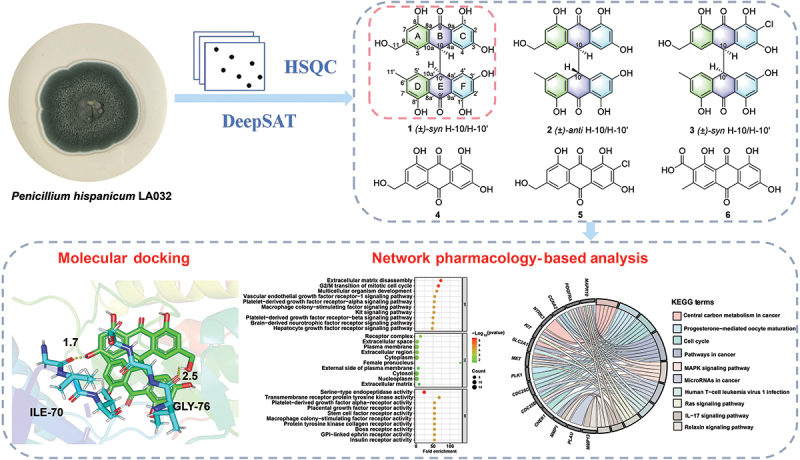

## 1. Introduction

Fungi serve as prolific sources of novel compounds with significant pharmacological activities, making them promising sources for drug lead development (Newman and Cragg 2020; Luo et al. 2024; Shi et al. 2024a). In particular, mangrove-derived fungi inhabiting tropical/subtropical coastal ecotones are emerging as prolific repositories for architecturally distinct novel compounds with a variety of bioactivities (Xu 2015; Chen et al. 2022; Zhu et al. 2022). Species of the genus *Penicillium* from mangrove ecosystems, harbouring diverse biosynthetic gene clusters, could produce structurally varied secondary metabolites, including alkaloids, terpenes, isocoumarins, and polyketides, with significant antimicrobial, cytotoxic, and anti-inflammatory activities (Meng et al. 2016; Liu et al. 2017; Qiu et al. 2020). The fungal species *Penicillium hispanicum* is in the *Ramigena* section of the *Ramigena* series (Houbraken et al. 2020). Up to now, reports of various compounds from *P. hispanicum* include the desoxyisoaustamide-type alkaloids, brevianamide-type alkaloid, anthraquinone and bisanthrone derivatives, together with some other polyketides (Houbraken et al. 2020; Nesterenko et al. 2023; Starnovskaya et al. 2024).

DeepSAT (https://deepsat.ucsd.edu) is a neural network-driven NMR-based structure annotation and scaffold prediction system designed to accelerate the identification of small molecules (Kim et al. 2023). Leveraging a convolutional neural network (CNN) architecture trained on a large number of experimentally collected or computationally derived HSQC spectra, DeepSAT directly extracts critical molecular features from input NMR spectra, such as chemical fingerprints, molecular weights, and structural classes (Kim et al. 2023; Wang et al. 2025). These features enable efficient database searches to identify structurally related compounds or analogs, even in the absence of authentic reference spectra (Duan et al. 2024; Liang et al. 2025). By integrating spectral interpretation with predictive scaffold analysis, DeepSAT enhances both the efficiency and accuracy of structure elucidation in drug discovery, offering a versatile solution to overcome bottlenecks in NMR-driven compound annotation and structural dereplication.

**Figure 1.**
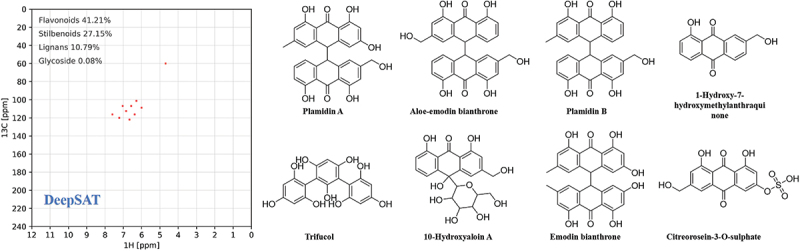
Digitised HSQC spectrum and the DeepSAT results.

**Figure 2.**
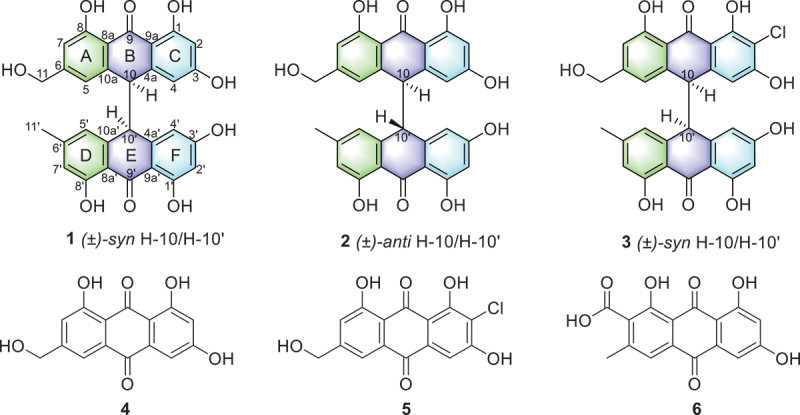
Chemical structures of compounds **1**−**6** (arbitrary enantiomers displayed for **1**−**3**).

In our ongoing studies to search for new bioactive compounds from the mangrove-derived fungi (Huo et al. 2022; Lu et al. 2024; Shi et al. 2024b), the strain *Penicillium hispanicum* LA032 was isolated from the mangrove rhizospheric soil and screened out for investigations. With the help of HSQC-based DeepSAT (Figure 1), three new racemic bianthrones, including two pairs of (±)-penithrones A (**1**) and B (**2**), and a chlorinated derivative (±)-penithrone C (**3**), along with anthraquinone derivatives, itreorosein (**4**) (Liang et al. 2012), 2-chloro-1,3,8-trihydroxy-6-(hydroxymethyl)anthracene-9,10-dione (**5**) (Yamamoto et al. 1968), and endocrocin (**6**) (Mai et al. 2001) (Figure 2) were discovered from the mangrove-derived fungus *P. hispanicum* LA032. Furthermore, these new compounds were assessed for their cytotoxic activities against HeLa, HCT116, and MCF-7 cancer cell lines. Since compound **1** exhibited significant inhibitory effects on multiple cancer cell lines, further network pharmacology analysis and molecular docking studies were conducted to elucidate its potential anticancer mechanisms of effect. Herein, we report the isolation, structural elucidation, and biological evaluation of these compounds, as well as the potential mechanism of action for its anticancer effect of compound **1**.

## 2. Materials and methods

### 2.1. General experimental procedures

The experimental methodology in this work was based on previous experimental procedures (Ren et al. 2021; Huo et al. 2022).

### 2.2. Fungal material

The strain LA032 was isolated from the source consistent with previous report (Huo et al. 2022). The strain was identified according to its phylogenetic analysis using ITS (GenBank Accession No. OP804236), CaM (GenBank Accession No. PV665029), BenA (GenBank Accession No. PV665028), and RPB2 (GenBank Accession No. PV665030) sequences, combined with morphological characterisation.

### 2.3. Fermentation, extraction, and DeepSAT-guided isolation

The fermentation and extraction processes were carried out in accordance with the previously reported methods (Huo et al. 2022). Its static fermentation was performed at 25 °C for 30 d. Finally, the crude extract was obtained (51.0 g). The extract underwent silica gel column chromatography (CC) with petroleum ether (PE)/EtOAc gradient elution, yielding eight fractions Fr.1–Fr.8.

Then a small quantity of fractions (25 mg) were taken into NMR tube, respectively. The HSQC experiments were performed through CSV data files uploaded to the DeepSAT system (https://deepsat.ucsd.edu). The result of Fr.3 indicated that six of top eight compounds, were bianthrones and anthraquinones (Figure 1). Based on the results of the DeepSAT positioning, Fr.3 could be rich in bianthrones and anthraquinones, thus it was systematically further investigated for its chemical constituents.

Fr.3 eluted with 80%−90% EtOAc were combined and separated via ODS CC (20%–100%; MeOH–H_2_O) to afford five subfractions Fr.3.1–Fr.3.5. Fr.3.2 subsequently underwent RP-HPLC purification (Reprosil-Pur Basic C-18 column; 250 × 10 mm; 5 μm; MeCN:H₂O, 55:45; 2.0 mL/min), yielding compounds **4** (2.1 mg, *t*_R_ 13.5 min) and **5** (1.5 mg, *t*_R_ 17.5 min). Purification of Fr.3.3 by RP-HPLC (Reprosil-Pur Basic C-18 column; 250 × 10 mm; 5 μm; MeOH/H_2_O, 30%–85%; 2.0 mL/min) provided compound **6** (2.0 mg, *t*_R_ 23.4 min). Further purification of Fr.3.4 yielded compound **3** (3.1 mg, *t*_R_ 30.0 min) using RP-HPLC (Reprosil-Pur Basic C-18 column; 250 × 10 mm; 5 μm; MeCN/H_2_O, 56:44; 2.0 mL/min). Fr.3.5 was further subjected to RP-HPLC (Reprosil-Pur Basic C-18 column; 250 × 10 mm; 5 μm; MeCN/H_2_O, 1:1; 2.0 mL/min), affording compounds **1** (3.4 mg, *t*_R_ 30.2 min) and **2** (2.5 mg, *t*_R_ 33.4 min).

(±)-Penithrone A (**1**): yellow solid; UV (MeOH) *λ*_max_ (log *ε*) 215 (2.74), 230 (2.70), 368 (2.46) nm; IR (neat) *ν*_max_ 3,117, 2,928, 1,619, 1,483, 1,337, 1,038 cm^−1^; HRESIMS at *m*/*z* 527.1331 [M + H]^+^ (calcd. for C_30_H_23_O_9_ *m*/*z* 527.1337).

(±)-Penithrone B (**2**): yellow solid; UV (MeOH) *λ*_max_ (log *ε*) 214 (2.83), 227 (2.79), 364 (2.55) nm; IR (neat) *ν*_max_ 3,076, 2,929, 1,621, 1,483, 1,382, 1,024 cm^−1^; HRESIMS at *m*/*z* 527.1338 [M + H]^+^ (calcd. for C_30_H_23_O_9_ *m*/*z* 527.1337).

(±)-Penithrone C (**3**): yellow solid; UV (MeOH) *λ*_max_ (log *ε*) 213 (2.78), 230 (2.51), 365 (2.33) nm; IR (neat) *ν*_max_ 3,394, 2,928, 1,604, 1,483, 1,373, 1,084 cm^−1^; HRESIMS at *m*/*z* 561.0947 [M + H]^+^ (calcd. for C_30_H_22_ClO_9_ *m*/*z* 561.0947).

### 2.4. Computational analysis

The NMR calculations for **1** and **2** were conducted by Gaussian 09 according to previously reported methods (Huo et al. 2022). The CP3 probabilities were then computed as originally described by Smith and Goodman according to previously reported methods (Kim et al. 2017).

### 2.5. Cytotoxic assays

Cytotoxic assays were performed as previously described (Liu et al. 2015). Detailed procedures were provided in the Supplementary files (experimental details and spectroscopic data of **1**−**3**).

### 2.6. Prediction and integration of compound 1 targets

The potential targets of compound **1** were predicted using multiple-platform databases, including SwissTargetPrediction (http://swisstargetprediction.ch/), Super-PRED (https://prediction.charite.de/), and PharmMapper (https://www.lilab-ecust.cn/pharmmapper/). RNA-seq data for breast cancer (TCGA-BRCA), colon cancer (TCGA-COAD), and cervical cancer (TCGA-CESC) were acquired from The Cancer Genome Atlas (TCGA). Furthermore, differentially expressed genes (DEGs) were determined using the DESeq2 R package based on criteria of adjusted *p*-value (padj) < 0.05 and absolute log2 fold change (|log2FC|) > 1. Volcano plots were generated using ggplot2 to visualise the DEGs. The overlapping targets between the compound **1** and cancer-specific DEGs (BRCA, COAD, and CESC) were analysed using the Venny 2.1 online tool.

### 2.7. Functional enrichment

The functional enrichment in this work was in accordance with those previously experimental procedures (Shi et al. 2024b). Further experimental details were available in the Supporting Information.

### 2.8. Molecular docking

The molecular docking in this work followed those previously reported (Shi et al. 2024b). Further experimental details were available in the Supporting Information.

## 3. Results and discussion

### 3.1. Identification of the strain

**Figure 3.**
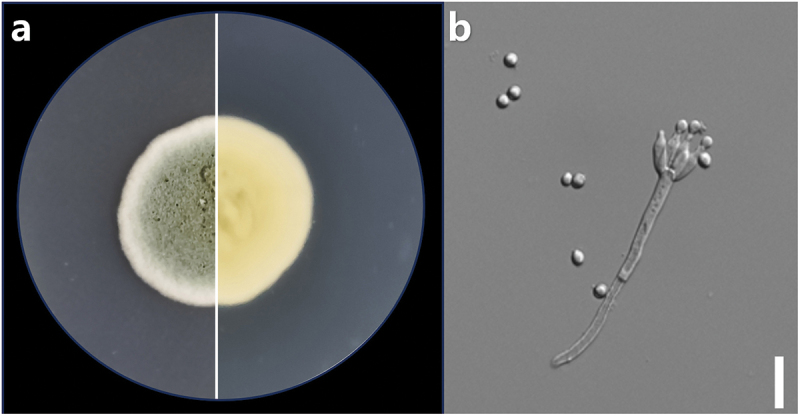
The phenotypical and morphological characters of *Penicillium hispanicum* LA032. (a) The colony. (b) Conidiophores and conidia. Scale bars: b = 2 µm.

**Figure 4.**
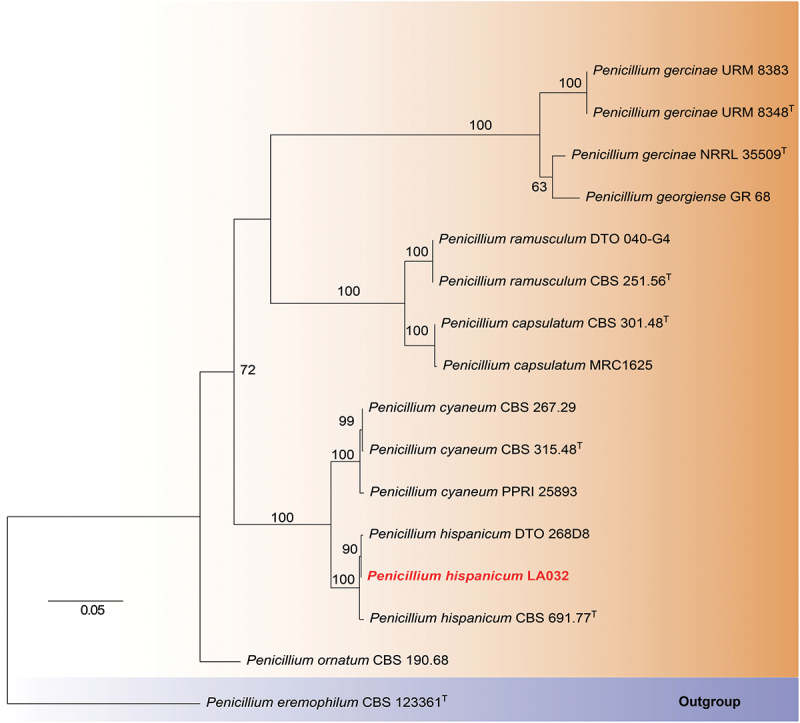
Maximum likelihood tree of *Penicillium* constructed using ITS, CaM, BenA, and RPB2 sequences. Bootstrap support values of maximum likelihood (MLBP) above 50% are shown at the nodes. The tree was rooted t*o Penicillium eremophilum* CBS 123361. The strain LA032 is printed in ped and bold font.

The fungus was identified by its phylogenetic analysis based on morphological observation and ITS, CaM, BenA, and RPB2 sequences (Figures 3 and 4). To determine the evolutionary position of the fungus LA032, a phylogenetic analysis was performed based on the ITS, CaM, BenA, and RPB2 sequences with those from other *Penicillium* species. The results showed that the strain LA032 was clustered with species in the genus *Penicillium* (*P. hispanicum*) with high confidence.

### 3.2. DeepSAT-guided isolation

The ethyl acetate extract was subjected to silica gel column chromatography to provide eight fractions (Fr.1−Fr.8). The HSQC-based DeepSAT analysis was employed for the rapid screening and prediction of natural product skeletal structures. The results indicated that six of top eight compounds were bianthrones and anthraquinones derivatives (Figure 1). Fr.3 was then selected for further purification, leading to the discovery of three new pairs of bianthrones racemates and their possible biosynthetic precursors.

### 3.3. Structural characterisation

Penithrone A (**1**) was obtained as a yellow solid. The molecular formula was determined to be C_30_H_22_O_9_ (twenty degrees of unsaturation) based on the HRESIMS data at *m*/*z* 527.1331 [M + H]^+^ (calcd. for C_30_H_23_O_9_ *m*/*z* 527.1337). The presence of hydroxy (3,117 cm^−1^) and carbonyl (1,619 cm^−1^) groups was revealed by the IR absorption bands. The 1D NMR (Table 1) together with the HSQC spectrum of **1**, revealed diagnostic resonances including one methyl (*δ*_H/C_ 2.21/21.4), one methylene (*δ*_H/C_ 4.50/62.1), two sp^3^ methines (*δ*_H/C_ 4.55/55.2; 4.49/55.1), eight aromatic methines (*δ*_H/C_ 6.17/101.6; 6.19/109.1; 6.51/117.6; 6.84/112.9; 6.21/101.6; 5.91/109.3; 6.07/121.2; 6.66/116.2), eighteen quarternary carbons, including two carbonyl groups (*δ*_C_ 189.5 and 189.5) and sixteen aromatic carbons (*δ*_C_ 151.2, 146.1, 143.7, 143.1, 141.1, 140.3, 114.7, 113.6, 109.3, 109.1; six oxygenated ones, *δ*_C_ 164.4, 164.3, 163.7, 163.6, 160.9, 160.9), and seven exchangeable protons (*δ*_H_ 5.37, 10.75, 10.79, 11.78, 11.86, 11.87, 11.90). The 1D and 2D NMR data of **1** showed similarities to those found in emodin bianthrone (Ji et al. 2014), exhibiting typical chemical shifts for the bianthrone ring system.

**Table 1.** ^1^H NMR (500 MHz) and ^13^C NMR data (125 MHz) for **1**–**3** in DMSO.

Pos.	1		2		3	
	*δ*_H_ (*J* in Hz)	*δ*_C_, mult.	*δ*_H_ (*J* in Hz)	*δ*_C_, mult.	*δ*_H_ (*J* in Hz)	*δ*_C_, mult.
1		164.3, C		164.4, C		158.9, C
2	6.17, d (2.0)	101.6, CH	6.24, d (2.0)	101.5, CH		105.9, CH
3		163.6, C		163.7, C		159.8, C
4	6.19, br s	109.1, CH	6.13, br s	109.2, CH	6.42, s	108.5, CH
4a		143.1, C		142.8, C		143.6, C
5	6.51, br s	117.6, CH	6.29, br s	117.6, CH	6.49, br s	117.5, CH
6		151.2, C		150.8, C		151.2, C
7	6.84, s	112.9, CH	6.79, s	113.0, CH	6.84, s	112.9, CH
8		160.9, C		160.9, C		160.9, C
8a		114.7, C		114.4, C		114.7, C
9		189.5, C		189.5, C		189.4, C
9a		109.3, C		109.3, C		109.7, C
10	4.55, d (2.0)	55.2, CH	4.56, d (2.0)	55.2, CH	4.56, d (2.0)	55.1, CH
10a		140.3, C		140.1, C		140.3, C
11	4.50, s	62.1, CH_2_	4.42, s	62.2, CH_2_	4.50, d (8.0)	62.2, CH_2_
1′		164.4, C		164.5, C		163.8, C
2′	6.21, d (2.0)	101.6, CH	6.22, d (2.0)	101.5, CH	6.18, d (2.0)	101.7, CH
3′		163.7, C		163.7, C		164.3, C
4′	5.91, br s	109.3, CH	6.13, br s	109.5, CH	5.93, br s	109.3, CH
4a′		141.1, C		141.2, C		142.9, C
5′	6.07, br s	121.2, CH	6.19, br s	121.1, CH	6.10, br s	121.3, CH
6′		146.1, C		146.1, C		146.7, C
7′	6.66, s	116.2, CH	6.67, s	116.3, CH	6.70, s	116.3, CH
8′		160.9, C		160.7, C		160.9, C
8a′		113.6, C		113.7, C		113.4, C
9′		189.5, C		189.5, C		189.4, C
9a′		109.1, C		109.4, C		109.0, C
10′	4.49, d (3.0)	55.1, CH	4.51, d (3.0)	55.2, CH	4.53, d (3.0)	54.8, CH
10a′		143.7, C		144.1, C		144.1, C
11′	2.21, s	21.4, CH_3_	2.23, s	21.4, CH_3_	2.22, s	21.5, CH_3_
OH-11	5.37, br s		5.34, br s		5.39, br s	
OH-1	10.74, s		10.82, s		12.55, s	
OH-3	11.87, s		11.92, s		11.56, s	
OH-8	11.86, s		11.78, s		11.86, s	
OH-1′	10.79, s		10.83, s		11.85, s	
OH-3′	11.90, s		11.95, s		10.81, s	
OH-8′	11.78, s		11.73, s		11.58, s	

**Figure 5.**
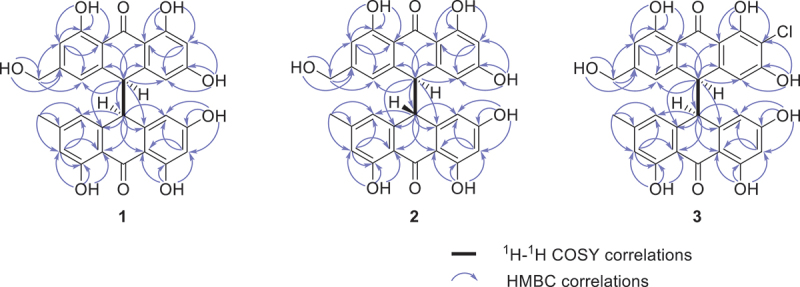
Selected 2D NMR correlations for racemates (arbitrary enantiomers displayed) of the *syn* isomer (±)-**1**, *anti* isomer (±)-**2**, and *syn* isomer (±)-**3**.

The planar structure of **1** was further determined by 2D NMR experiments (Figure 5). The HMBC correlations from OH-1 (*δ*_H_ 10.74) to the oxygenated carbon C-1 (*δ*_C_ 164.3), the aromatic methine carbon C-2 (*δ*_C_ 101.6), and C-9a (*δ*_C_ 109.3), from OH-3 (*δ*_H_ 11.87) to C-2, the oxygenated carbon C-3 (*δ*_C_ 163.6), and C-4 (*δ*_C_ 109.1), from the aromatic proton H-2 (*δ*_H_ 6.17) to C-4 and C-9a, from OH-8 (*δ*_H_ 11.86) to the aromatic methine C-7 (*δ*_C_ 112.9), the oxygenated carbon C-8 (*δ*_C_ 160.9), and C-8a (*δ*_C_ 114.7), from the aromatic proton H-7 (*δ*_H_ 6.84) to C-5 (*δ*_C_ 117.6) and C-8a, and from the sp^3^ methine H-10 (*δ*_H_ 4.55) to C-4, C-5, C-4a (*δ*_C_ 143.1), C-8a, C-9a, and C-10a (*δ*_C_ 140.3) allowed for the construction of the anthrone moiety (rings A−C) with three hydroxy groups located at positions C-1, C-3, and C-8, respectively. Further HMBC correlations from the oxygenated methylene H_2_-11 (*δ*_H_ 4.50) to C-5, C-6 (*δ*_C_ 151.2), and C-7, and from OH-11 (*δ*_C_ 5.37) to C-11 (*δ*_C_ 62.1) and C-6, indicated a hydroxymethyl group (CH_2_-11) attached at C-6. Similarly, the HMBC correlations from OH-1′ (*δ*_H_ 10.79) to the oxygenated carbon C-1′ (*δ*_C_ 164.4), the aromatic methine carbon C-2′ (*δ*_C_ 101.6), and C-9a′ (*δ*_C_ 109.1), from OH-3′ (*δ*_H_ 11.90) to C-2′, the oxygenated carbon C-3′ (*δ*_C_ 163.7), and C-4′ (*δ*_C_ 109.3), from the aromatic proton H-2′ (*δ*_H_ 6.21) to C-4 and C-9a, from OH-8′ (*δ*_H_ 11.78) to the aromatic methine carbon C-7′ (*δ*_C_ 116.2), C-8′ (*δ*_C_ 160.9), and C-8a′ (*δ*_C_ 113.6), from H-7′ (*δ*_H_ 6.66) to C-5′ (*δ*_C_ 121.2) and C-8a′, from the sp^3^ methine H-10′ (*δ*_H_ 4.49) to C-4a′ (*δ*_C_ 141.1), C-8a′, C-9a′, and C-10a′ (*δ*_C_ 143.7), and from the methyl H-11′ (*δ*_H_ 2.21) to C-5′, C-6′ (*δ*_C_ 146.1), and C-7′, established the another anthrone moiety (rings D−F) with three hydroxy groups and one methyl located at positions C-1′, C-3′, C-8′, and C-6′, respectively. Moreover, the ^1^H–^1^H COSY cross peak of H-10/H-10′ combined with the key HMBC correlations from H-10 to C-10′, C-4a′ and C-10′, and from H-10′ to C-4a, C-10 and C-10a, confirmed the connection the two anthrone moiety via a C-10′–C-10 linkage. Therefore, the structure of **1** was elucidated as depicted in Figure 2. The specific rotation value of **1** was zero, which indicated that **1** was a racemate. However, chiral-phase resolution using different conditions failed.

HRESIMS analysis of **2** revealed the molecular formula C_30_H_22_O_9_, suggestive of an isomer of **1**. A detailed 1D and 2D NMR data of **1** and **2** showed slight differences in the chemical shifts. The combined analysis of ^1^H-^1^H COSY and HMBC spectra (Figure 5) revealed that compound **2** has the same planar structure as compound **1**. The small specific rotation value revealed that **2** was also a racemate. Despite extensive efforts employing various conditions, chiral-phase resolution was unsuccessful.

**Figure 6.**
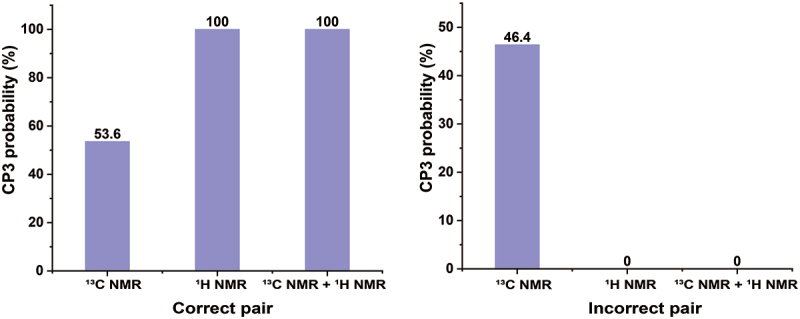
CP3 probability analysis.

In order to confirm the relative configurations of **1** and **2**, CP3 analyses were performed. The CP3 metric effectively identifies subtle NMR chemical shift differences between isomers by comparing experimental and calculated data, offering a higher sensitivity than the DP4 method, which relies on minimising absolute deviations (Smith and Goodman 2009; Kim et al. 2017). While DP4 prioritises matching calculated shifts to experimental values for individual diastereomers, CP3 focuses on analysing relative shielding variations across isomers. This allows CP3 to better evaluate magnetic shielding trends and distinguish the candidate isomers (Smith and Goodman 2009; Kim et al. 2017; Kanehara et al. 2024). As shown in Figure 6, the CP3 analysis supported the NMR-based assignments produced for correct pair (*syn* isomer for **1** and *anti* isomer for **2**) with 100% probability when both the ^1^H and ^13^C NMR chemical shift values were considered. Thus, compounds **1** and **2** were identified as the new *syn* isomer (±)-penithrone A (**1**) and the new *anti* isomer (±)-penithrone B (**2**), respectively.

The molecular formula of **3** was assigned to be C_30_H_21_ClO_9_ on the basis of the HRESIMS [M + H]^+^ *m*/*z* 561.0947 (calcd. for C_30_H_22_ClO_9_ *m*/*z* 561.0947). The isotopic ion peak was observed at *m/z* 563.0928 [M + 2 + H]^+^ in the ratio of 3:1, indicating the existence of a chlorine atom in **3**. The ^13^C NMR spectrum (Table 1) revealed the presence of 30 carbon signals including one methyl, one oxygenated methylene, two sp^3^ methines, seven aromatic methine carbons, nineteen quarternary sp^2^ carbons including two carbonyl carbons and six oxygenated carbons. The NMR spectroscopic data of **3** were similar to those of **1**, except for an aromatic proton at C-7 in **1** which was replaced by a chlorine atom, as confirmed by the HMBC correlations (Figure 5) from OH-6 and OH-8 to C-7. Thus, the planar structure of compound **3** was established. The absence of a specific rotation confirmed **3** to exist as a racemic mixture. Nevertheless, attempts at chiral-phase resolution under diverse experimental conditions proved to be unsuccessful. Determining the relative configuration of **3** could be conducted by the comparison of the ^1^H NMR of **3** with those of **1** and **2**. The ^1^H NMR spectrum of compound **3** was more similar to those of compound **1** (Table 1) than to those of compound **2** (Ji et al. 2014), allowing the relative configuration of **3** to be established as the new *syn* isomer (±)-penithrone C (**3**).

Bianthrones, generated by the fusion of two anthrone precursors, are rare natural compounds found in plants and fungi, typically substituted with methyl, hydroxy, methoxy, or occasionally more complex groups (Ji et al. 2014; Lin et al. 2021). These compounds exhibit significant biological activities, including cytotoxicity (Mandelare et al. 2018, 2024), antimicrobial activity (Elbanna et al. 2021; Freire et al. 2024), and antiplasmodial effects (Ndjakou Lenta et al. 2008). The elucidation of relative configurations at positions 10 and 10′ presents significant challenges in bianthrones (Ji et al. 2014; Elbanna et al. 2021). To date, the used methods for determining the relative configuration of bianthrones mainly include: ^1^H NMR chemical shift analysis (Ji et al. 2014), HPLC retention times (Elbanna et al. 2021), and CP3 analysis (Form et al. 2019; Lagarde et al. 2021). The HPLC retention time approach has proven effective in distinguishing *trans* from *cis* bianthrone isomers (Elbanna et al. 2021). However, its current applicability was limited by reliance on analyses conducted with C-8 columns. The most widely utilised methods were ^1^H NMR chemical shift analysis and the CP3 parameter evaluation. While ^1^H NMR chemical shift analysis provided rapid insights into relative configurations, it ultimately still needs to be verified by CP3 analysis.

The structures of three known compounds, citreorosein (**4**) (Liang et al. 2012), 2-chloro-1,3,8-trihydroxy-6-(hydroxymethyl)anthracene-9,10-dione (**5**) (Yamamoto et al. 1968), and endocrocin (**6**) (Mai et al. 2001), was established as their spectroscopic data showed complete consistency with literature.

### 3.4. Cytotoxic activity

Compounds **1**−**3** were evaluated for cytotoxic activity *in vitro* (Table 2) against human cancer lines HeLa, HCT116, and MCF-7. Compound **1** exhibited significant cytotoxic activities against HeLa, HCT116, and MCF-7 cell lines with IC_50_ values of 5.09 ± 0.65, 5.31 ± 0.48, and 5.23 ± 0.54 μmol/L, respectively, which are better than those of cisplatin (a positive control with IC_50_ values of 8.26 ± 1.83, 9.41 ± 1.85, and 9.00 ± 1.94 μmol/L, respectively). Compound **2** demonstrated cytotoxic activities against all tested cell lines with IC_50_ values of 5.42 ± 1.04, 8.86 ± 0.49, and 9.47 ± 0.22 μmol/L, respectively, comparable to those of cisplatin. Compound **3** also displayed modest cytotoxic activity against HeLa cell line with IC_50_ value of 17.36 ± 4.10 μmol/L.

**Table 2.** Cytotoxic effects of compounds **1**−**3**.

Compounds	Cell lines (μmol/L)		
	HeLa	HCT116	MCF7
**1**	5.09 ± 0.65	5.31 ± 0.48	5.23 ± 0.54
**2**	5.42 ± 1.04	8.86 ± 0.49	9.47 ± 0.22
**3**	17.36 ± 4.10	>50	42.13 ± 7.13
**Cisplatin**	8.26 ± 1.83	9.41 ± 1.85	9.00 ± 1.94

### 3.5. The potential targets and signalling pathways of compound 1 using network pharmacology analysis

**Figure 7.**
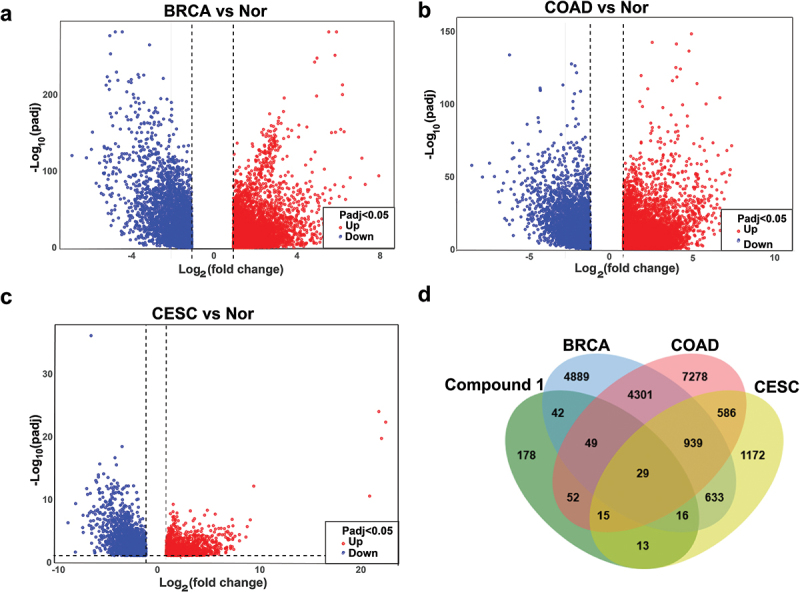
Results of target prediction and DEGs filtered. (a) Volcano map of DEGs in TCGA-BRCA. (b) Volcano map of DEGs in TCGA-COAD. (c) Volcano map of DEGs in TCGA-CESC. (d) Venn diagram results of compound **1** and cancer-specific DEGs.

**Figure 8.**
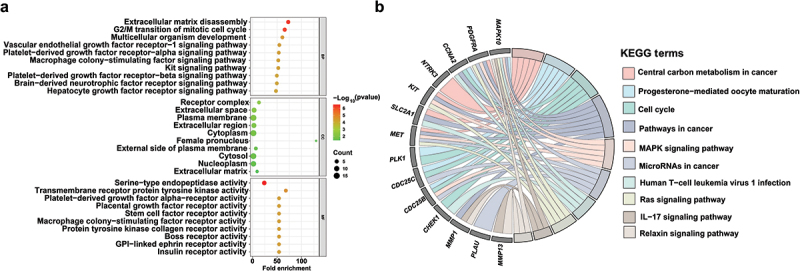
Functional enrichment analysis of common targets. (a) Go enrichment analysis of common targets. (b) KEGG enrichment analysis of common targets.

To further explore potential anticancer mechanism of compound **1**, network pharmacology analyses were performed. Computational target prediction revealed 394 potential target genes of compound **1** using SwissTargetPrediction, Super-PRED, and PharmMapper databases. Meanwhile, differential gene expression analysis of breast cancer (TCGA-BRCA), colon cancer (TCGA-COAD), and cervical cancer (TCGA-CESC) by TCGA transcriptomic datasets identified 10,965 BRCA (Figure 7a), 13,375 COAD (Figure 7b), and 3,404 CESC (Figure 7c) DEGs, respectively. Through the Venn diagram, the predicted targets and cancer-specific DEGs were intersected to yield 29 overlapping genes (*ABCG2*, *BLM*, *NTRK3*, *CDC25C*, *SCN4A*, *SCD*, *SLC2A1*, *PDGFRA*, *GRIA2*, *CDC25B*, *AOC3*, *CRABP2*, *MET*, *MMP1*, *CHEK1*, *PLAU*, *MAPK10*, *HSD17B11*, *ADH1B*, *BST1*, *PLK1*, *CBS*, *CFB*, *CMA1*, *MMP12*, *KIT*, *MMP13*, *CTSG*, and *CCNA2*) (Figure 7d). Subsequently GO and KEGG pathway analyses were conducted to elucidate the biological roles of these 29 candidate genes. GO analysis categorises the functions of DEGs systematically across three aspects: biological process, molecular function, cellular component. Top 10 enriched terms (*p* < 0.05) highlighted critical processes, including G2/M transition of the mitotic cell cycle and vascular endothelial growth factor receptor-1 signalling pathway (Figure 8a). KEGG analysis identified significantly enriched biological pathways among DEGs, revealing that significant enrichment was observed in cancer-related pathways, including central carbon metabolism, cell cycle, and MAPK/Ras signalling. Notably, *MAPK10* emerged as a central hub gene, regulating 70% of the enriched pathways (Figure 8b). Within the MAPK signalling cascade, MAPK10 regulates downstream transcription factors (e.g. JunD and ATF2), thereby modulating critical biological processes such as cell proliferation, differentiation, and apoptosis. Meanwhile, MAPK10 is also involved in regulating immune cell infiltration and angiogenesis within the tumour microenvironment (Zhang et al. 2019; Li et al. 2021), suggesting its potential therapeutic relevance. Therefore, MAPK10 could be a potential therapeutic target for compound **1** in cancer treatment.

### 3.6. Molecular docking validation of the potential target of compound 1

**Figure 9.**
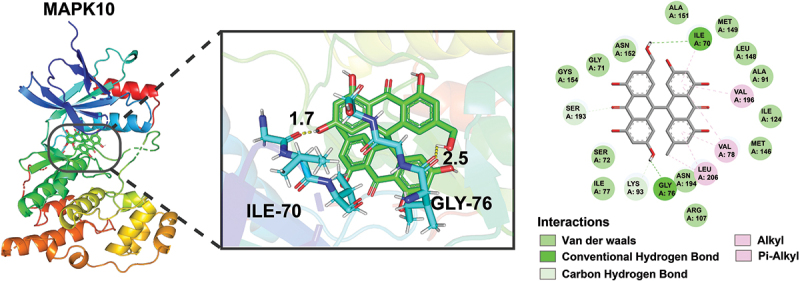
Docking result of compound **1** with MAPK10 (PDB: 7ksk).

Given the identification of MAPK10 as a key target of compound **1** (Figure 8b), molecular docking studies were further performed to explore their binding interactions between compound **1** and MAPK10. The docking results revealed that compound **1** exhibited a strong binding affinity (binding energy: −9.0 kcal/mol) with MAPK10. Further analysis revealed that compound **1** binds to MAPK10 by forming hydrogen bonds with key amino acid residues ILE70, GLY76, SER193, and LYS93. In addition, hydrophobic interactions are observed with VAL78, VAL196, and LEU206 (Figure 9). These observed molecular interactions between compound **1** and MAPK10 further validating MAPK10 as a potential target of compound **1** for its cytotoxic effect. These findings will provide a structural basis for further experimental validation and optimisation of compound **1** as a potential MAPK10 targeted therapeutic agent.

## 4. Conclusions

In summary, HSQC-based DeepSAT strategy was employed to target isolation of three new pairs of racemic bianthrones and chlorinated bianthrones (±)-penithrones A−C (**1**−**3**), along with their building blocks **4**−**6** from the mangrove-derived fungus *Penicillium hispanicum* LA032. Compound **1** showed significant cytotoxic effect against three human cancer cell lines: HeLa, HCT116, and MCF-7, surpassing the efficacy of cisplatin. Subsequently, network pharmacology and molecular docking analyses identified MAPK10 as the potential target of compound **1**. Future studies should focus on structural optimisation and mechanistic validation of these compounds. This study not only expands the structural diversity of bianthrone derivatives but also offers a molecular framework for lead compound development.

## Supplementary Material

Supplementary files.docx
