# Supplementary material for: Discovery of new bianthrones and chlorinated bianthrones with cytotoxic activity against cancer cells from Penicillium hispanicum guided by HSQC-based DeepSAT
Source: Mycology. 2025 Jul 10;17(1):2526766. doi: 10.1080/21501203.2025.2526766 (PMC13007469; doi:10.1080/21501203.2025.2526766)
Supplement: Supplementary files.docx [file TMYC_A_2526766_SM4565.docx]

Supplemental files

**Discovery of new bianthrones and chlorinated bianthrones with cytotoxic activity against cancer cells from *Penicillium hispanicum* guided by HSQC-based DeepSAT**

Ruiyun Huo^a,b,#^, Minhui Ji^a,b,#^, Gaoran Liu^a,b^, Ling Liu^a,b,*^

^a^State Key Laboratory of Microbial Diversity and Innovative Utilization, Institute of Microbiology, Chinese Academy of Sciences, Beijing, China

^b^University of Chinese Academy of Sciences, Beijing, China

^#^These authors contributed equally to this work.

^*^Corresponding author: Ling Liu, liul@im.ac.cn

**Table of contents**

| **Experimental section** | S1 |
| --- | --- |
| **General experimental procedures** | S1 |
| **Cell survival assay** | S1 |
| **Functional enrichment** | S1 |
| **Molecular docking** | S1 |
| **Figure S1.** HRESIMS spectrum of penithrone A (**1**). | S2 |
| **Figure S2.** ^1^H NMR spectrum of penithrone A (**1**; 500 MHz, DMSO). | S2 |
| **Figure S3.** ^13^C NMR spectrum of penithrone A (**1**; 125 MHz, DMSO). | S3 |
| **Figure S4.** HSQC spectrum of penithrone A (**1**; 500 MHz, DMSO). | S3 |
| **Figure S5.** HMBC spectrum of penithrone A (**1**; 500 MHz, DMSO). | S4 |
| **Figure S6.** HRESIMS spectrum of penithrone B (**2**). | S4 |
| **Figure S7.** ^1^H NMR spectrum of penithrone B (**2**; 500 MHz, DMSO). | S5 |
| **Figure S8.** ^13^C NMR spectrum of penithrone B (**2**; 125 MHz, DMSO). | S5 |
| **Figure S9.** HSQC spectrum of penithrone B (**2**; 500 MHz, DMSO). | S6 |
| **Figure S10.** HMBC spectrum of penithrone B (**2**; 500 MHz, DMSO). | S6 |
| **Figure S11.** HRESIMS spectrum of penithrone C (**3**). | S7 |
| **Figure S12.** ^1^H NMR spectrum of penithrone C (**3**; 500 MHz, DMSO). | S7 |
| **Figure S13.** ^13^C NMR spectrum of penithrone C (**3**; 125 MHz, DMSO). | S8 |
| **Figure S14.** HSQC spectrum of penithrone C (**3**; 500 MHz, DMSO). | S8 |
| **Figure S15.** HMBC spectrum of penithrone C (**3**; 500 MHz, DMSO). | S9 |
| **Figure S16**. The optimized conformers in NMR calculation for *syn* isomer and *anti* isomer. | S9 |

# Experimental section

**General experimental procedures**

The ECD spectra were obtained on Chirascan V100 instrument (Applied Photophysics Ltd, Leatherhead, Surrey, UK). Optical rotations were measured with an Anton Paar MCP 200 Automatic Polarimeter (Anton Paar, Graz, Austria). UV data were recorded using a Thermo Genesys-10S UV/Vis spectrophotometer (Thermo Fisher Scientific, Waltham, MA, USA). IR data were obtained on a Nicolet IS5 FT-IR spectrophotometer (Thermo Fisher Scientific, Waltham, MA, USA). ^1^H and ^13^C NMR data were acquired with Bruker Avance-500 spectrometer (Bruker, Bremen, Germany) using solvent signals (DMSO: *δ*_C/H_ 39.51/2.50) as reference. Mass data were performed on an Agilent Accurate-Mass-Q-TOF LC/MS 6520 instrument (Agilent Technologies, Santa Clara, CA, USA). HPLC separations were performed on an Agilent 1260 instrument equipped with a semipreparative Reprosil-Pur Basic C-18 column (250 × 10 mm, 5 μm, Dr Maisch HPLC GmbH, Beim Bruckle 14, Germany).

**Cell survival assay**

Briefly, cells were seeded into 96-well plates at a density of 5 × 10³ cells/well. After 24 h, cells were exposed to different concentrations of test compounds in medium supplemented with 10% fetal bovine serum and incubated for the indicated duration. The control cells were treated with the same volumes of DMSO. The cells were incubated with 100 μL of medium containing 20 μL of MTS at 37 °C for 1 h, and absorbance was measured at 490 nm using a Multi-Mode microplate reader.

**Functional enrichment**

Functional enrichment analysis, including Gene Ontology (GO) and Kyoto Encyclopedia of Genes and Genomes (KEGG) pathway analyses, was performed using the DAVID database. For GO analysis, the top 10 significantly enriched terms (*P* < 0.05) in the biological process (BP), molecular function (MF), and cellular component (CC) categories were selected by ranking gene counts in descending order. For KEGG pathway analysis, the top 20 enriched pathways (*P* < 0.05) were visualized.

**Molecular docking**

Molecular docking was conducted by AutoDock Vina with default parameters, and the crystal structure of MAPK10 protein (PDB: 7ksk) was obtained from the RCSB Protein Data Bank (http://www.wwpdb.org). The pdb file of MAPK10 and the compound SDF file were preprocessed using Open Babel. The docking result was visualized by PyMOL. Subsequent survival analysis was conducted using the GEPIA2 database (GEPIA2, https://gepia2.cancer-pku.cn/).


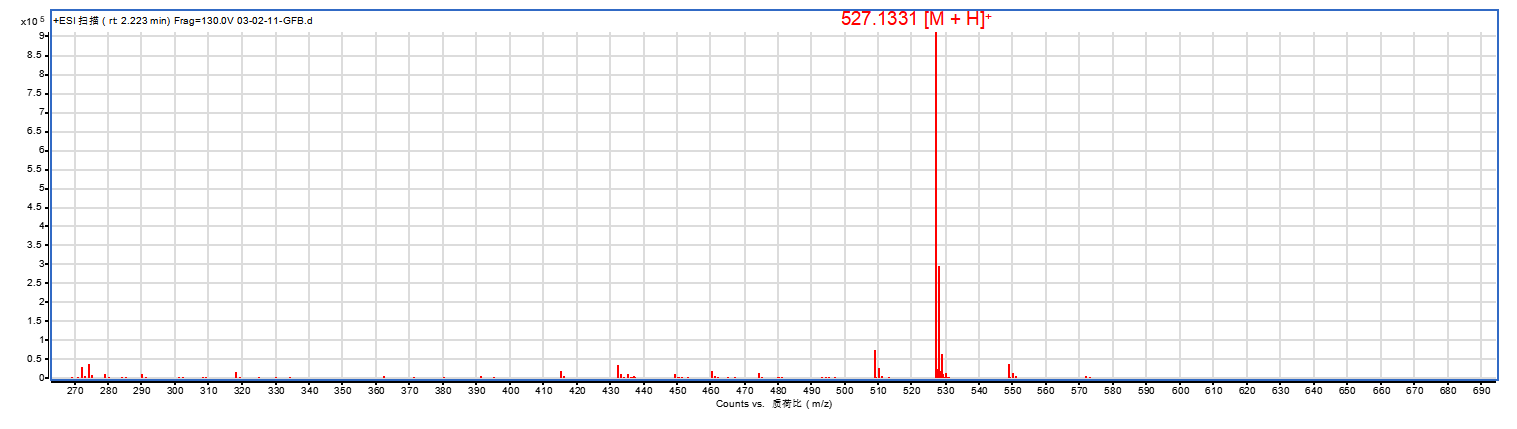


**Figure S1.** HRESIMS spectrum of penithrone A (**1**).


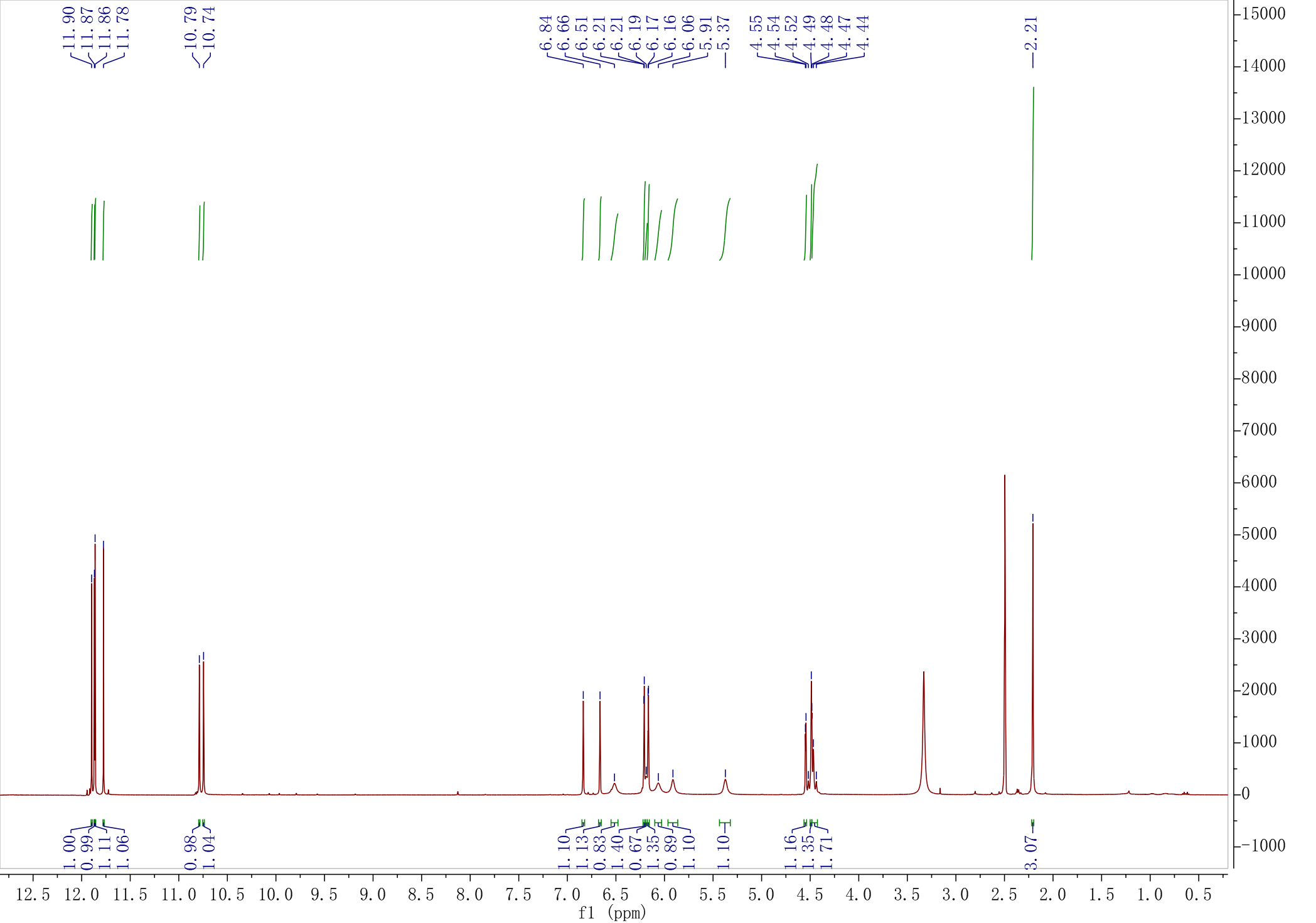


**Figure S2**. ^1^H NMR spectrum of penithrone A (**1**; 500 MHz, DMSO).


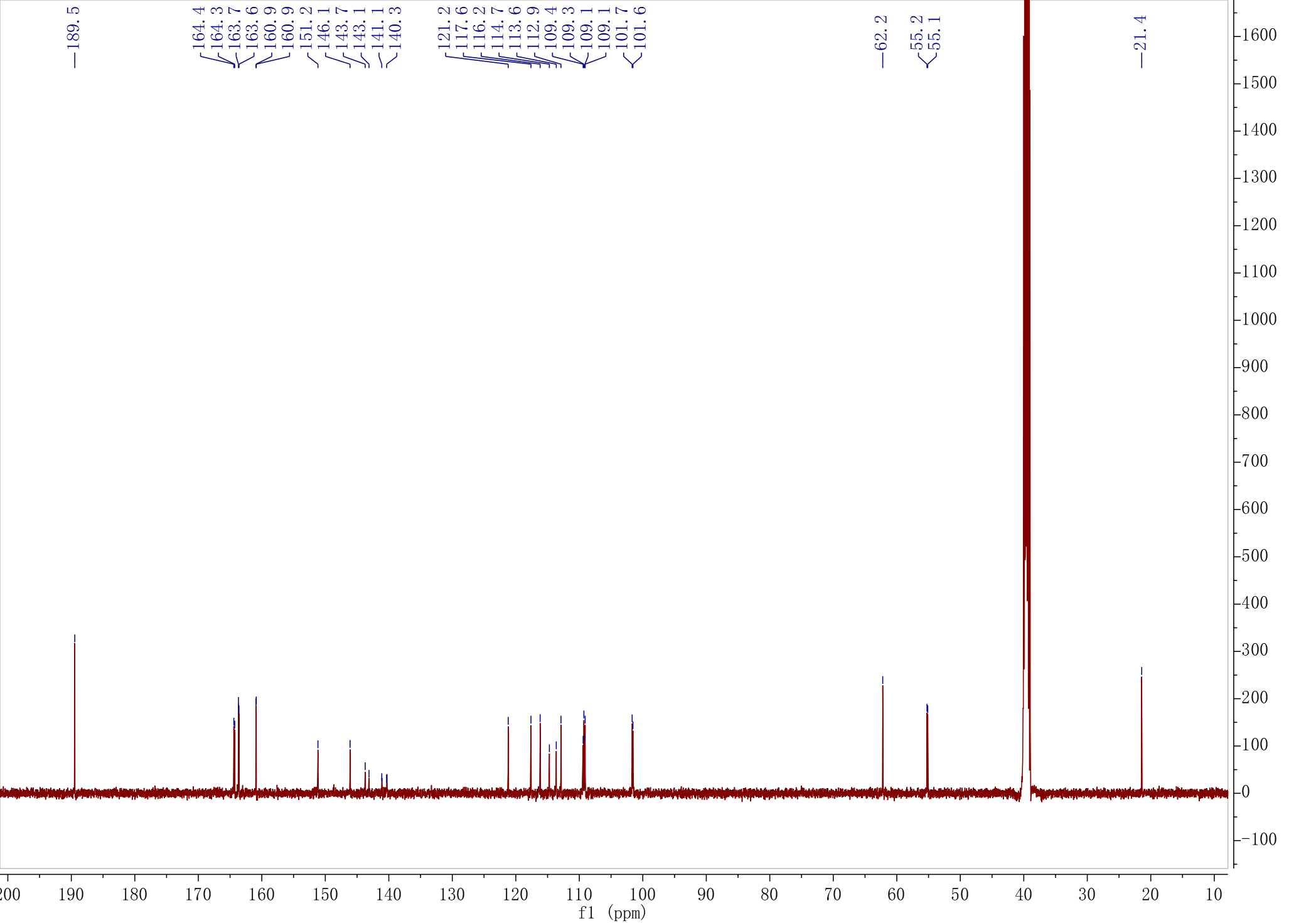


**Figure S3**. ^13^C NMR spectrum of penithrone A (**1**; 125 MHz, DMSO).


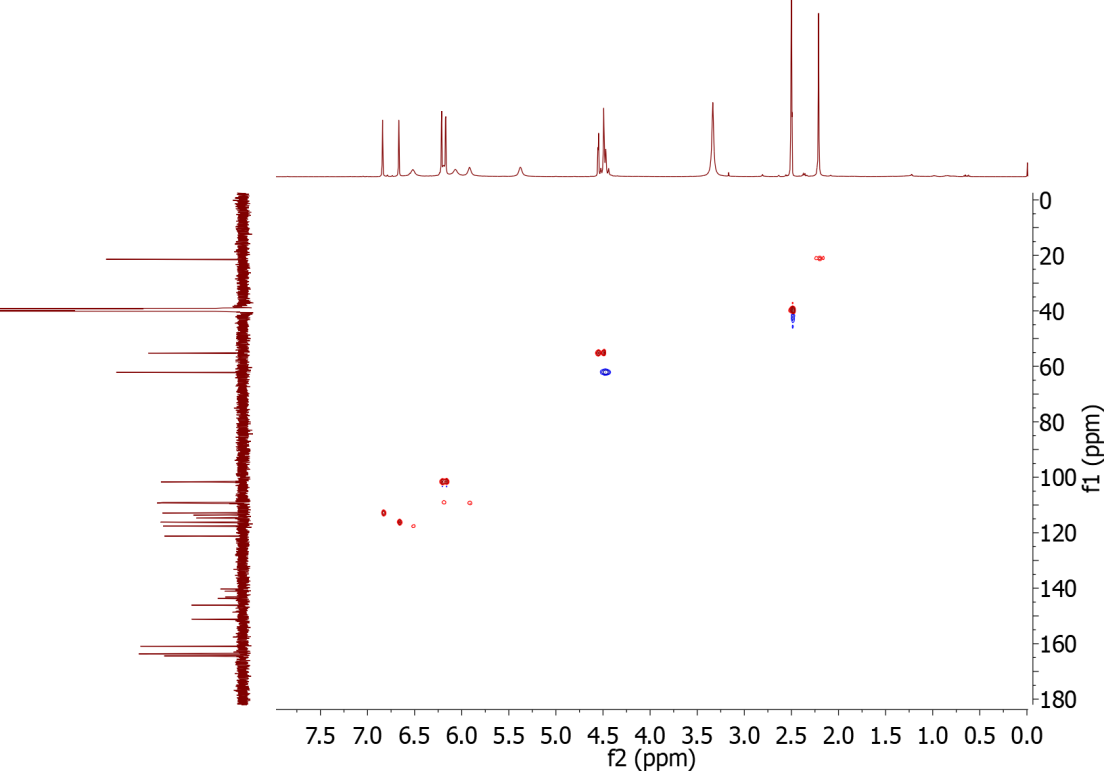


**Figure S4**. HSQC spectrum of penithrone A (**1**; 500 MHz, DMSO).


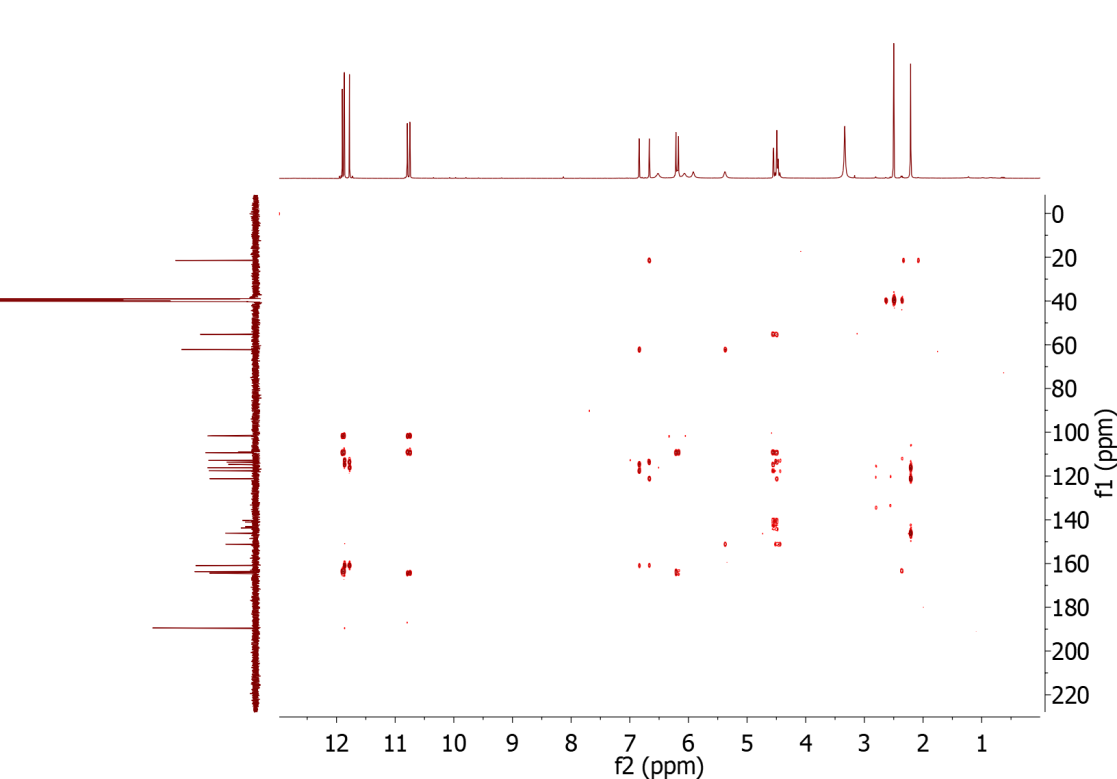


**Figure S5**. HMBC spectrum of penithrone A (**1**; 500 MHz, DMSO).


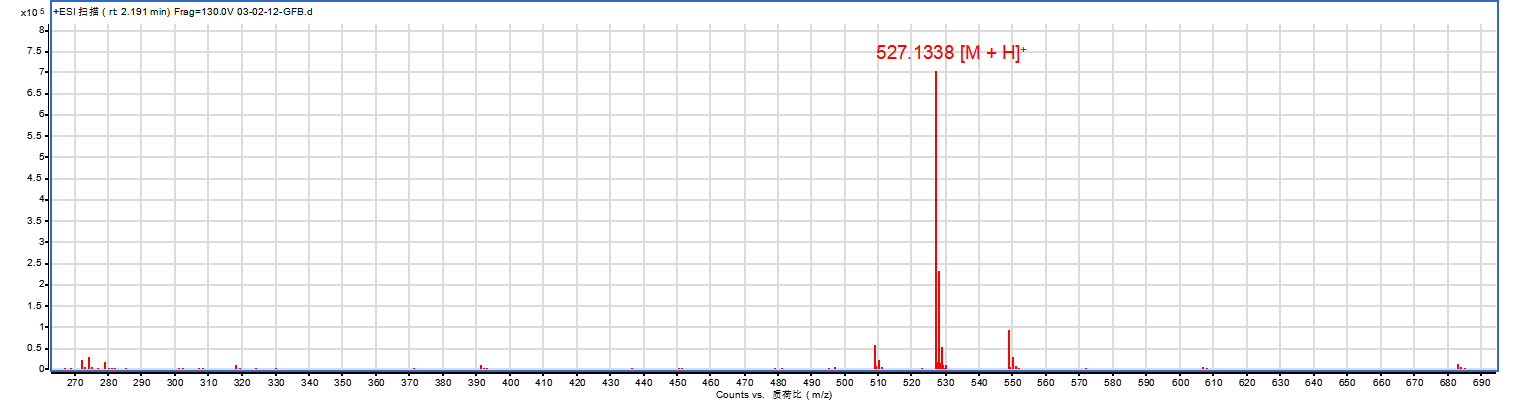


**Figure S6.** HRESIMS spectrum of penithrone B (**2**).


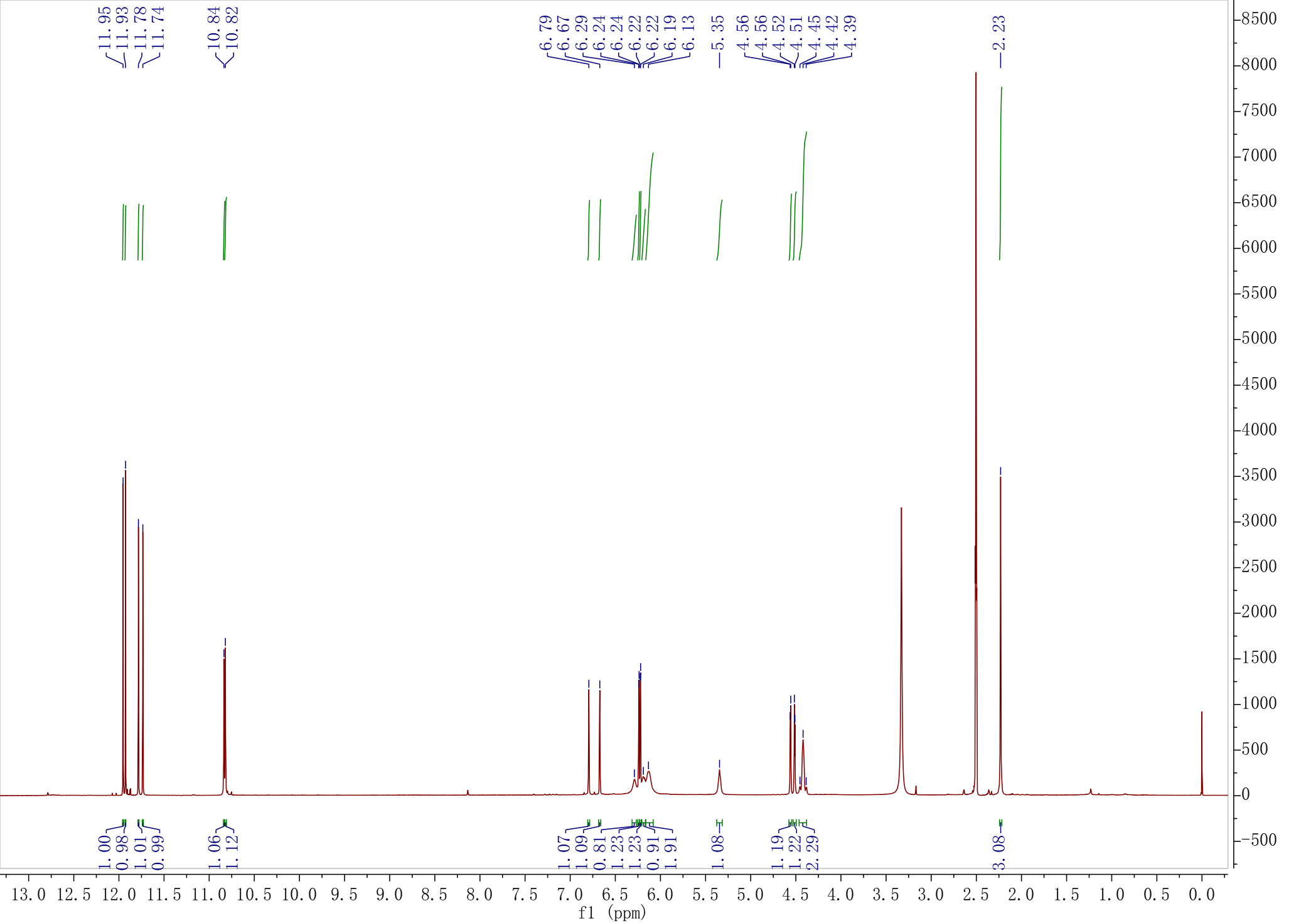


**Figure S7**. ^1^H NMR spectrum of penithrone B (**2**; 500 MHz, DMSO).


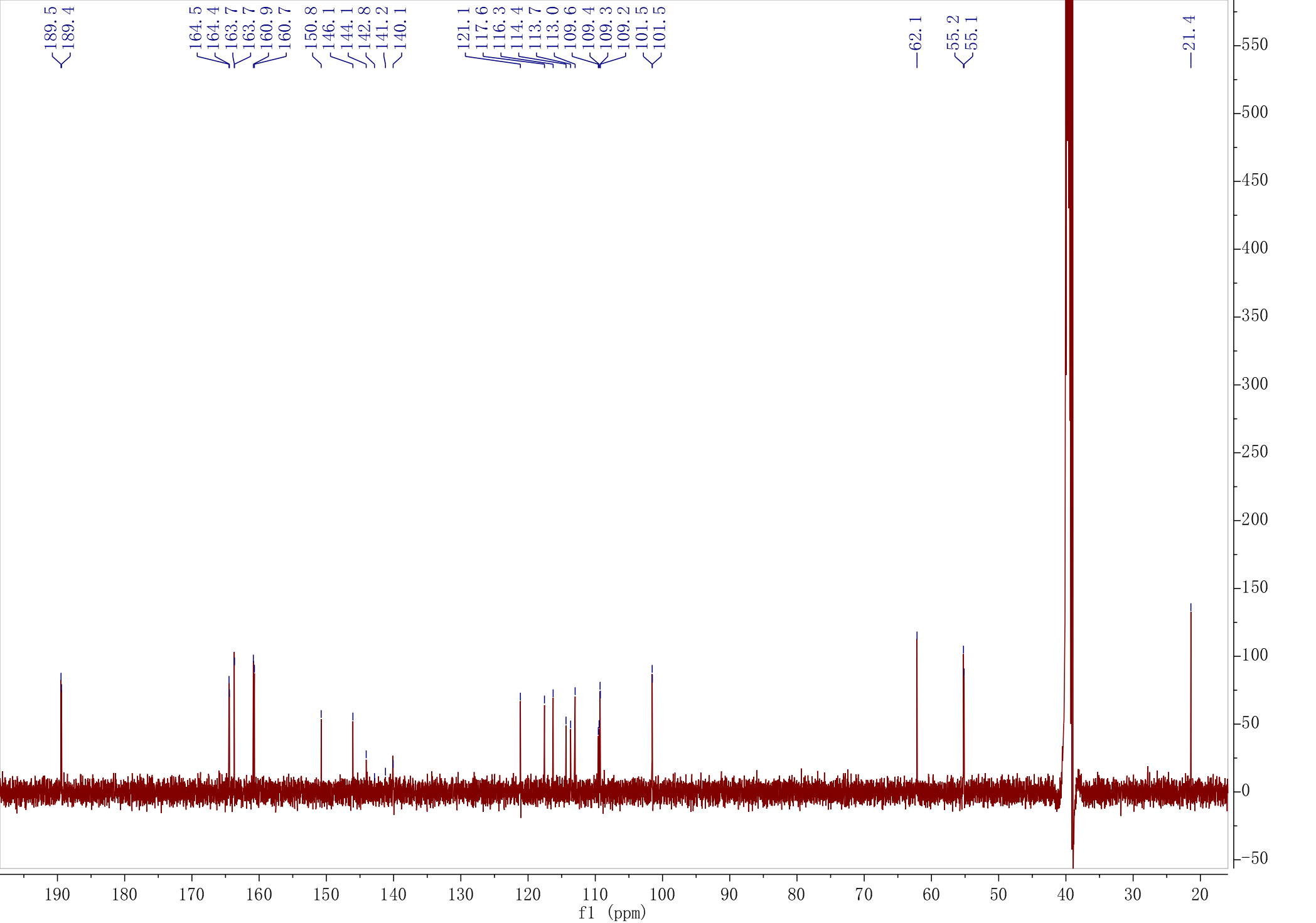


**Figure S8**. ^13^C NMR spectrum of penithrone B (**2**; 125 MHz, DMSO).


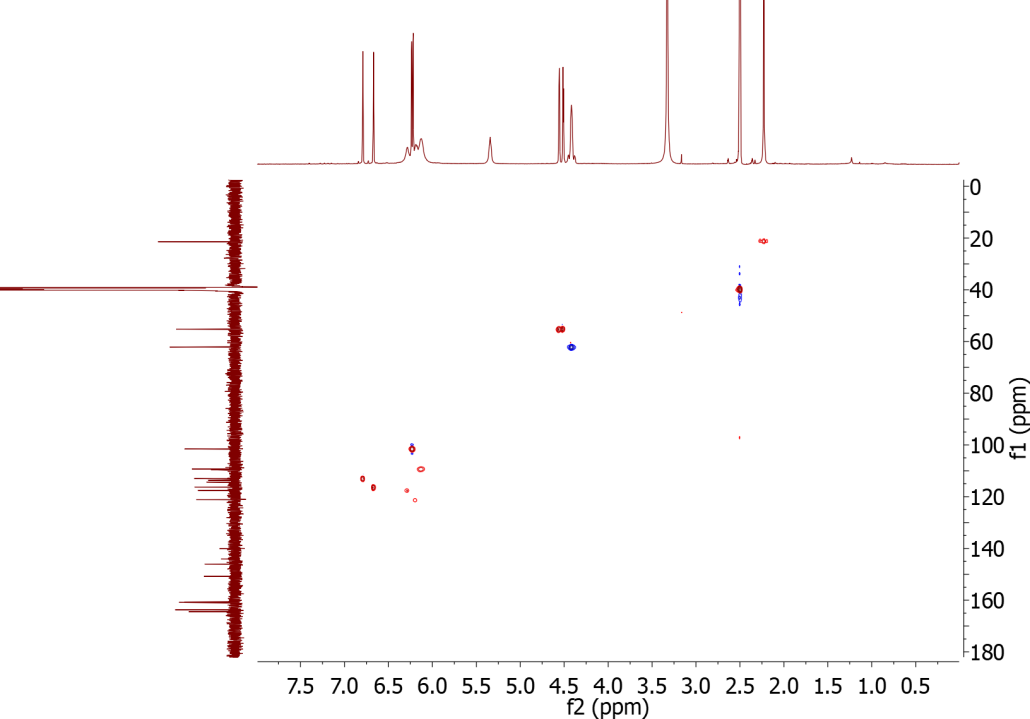


**Figure S9**. HSQC spectrum of penithrone B (**2**; 500 MHz, DMSO).


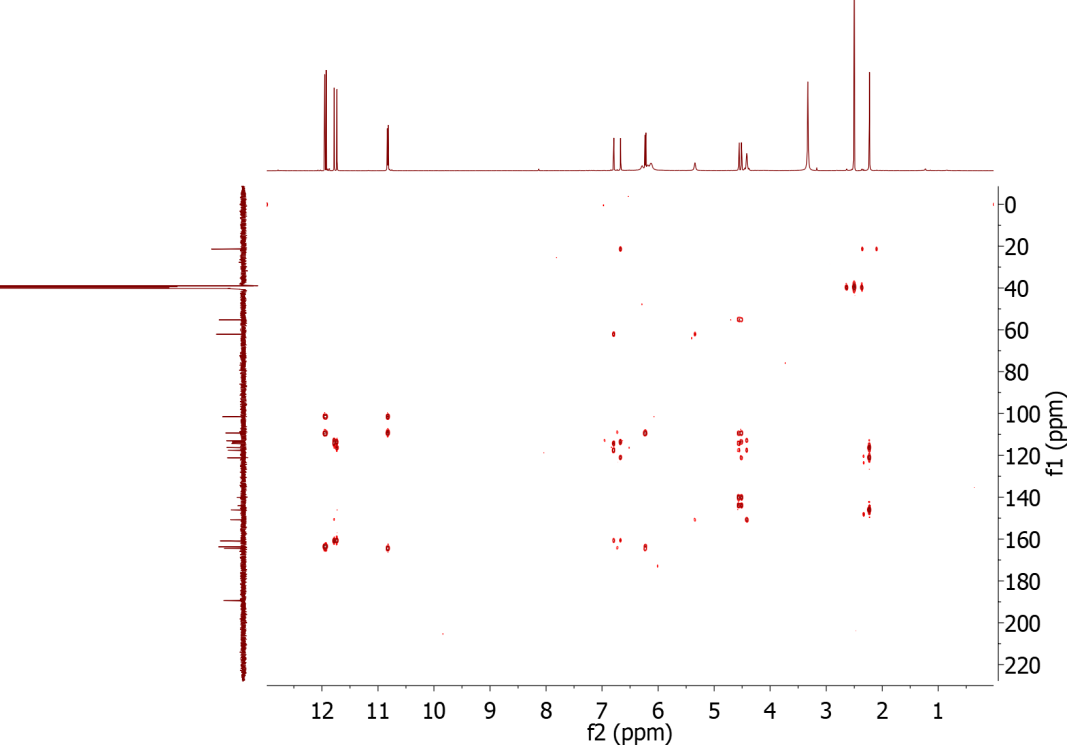


**Figure S10**. HMBC spectrum of penithrone B (**2**; 500 MHz, DMSO).

**
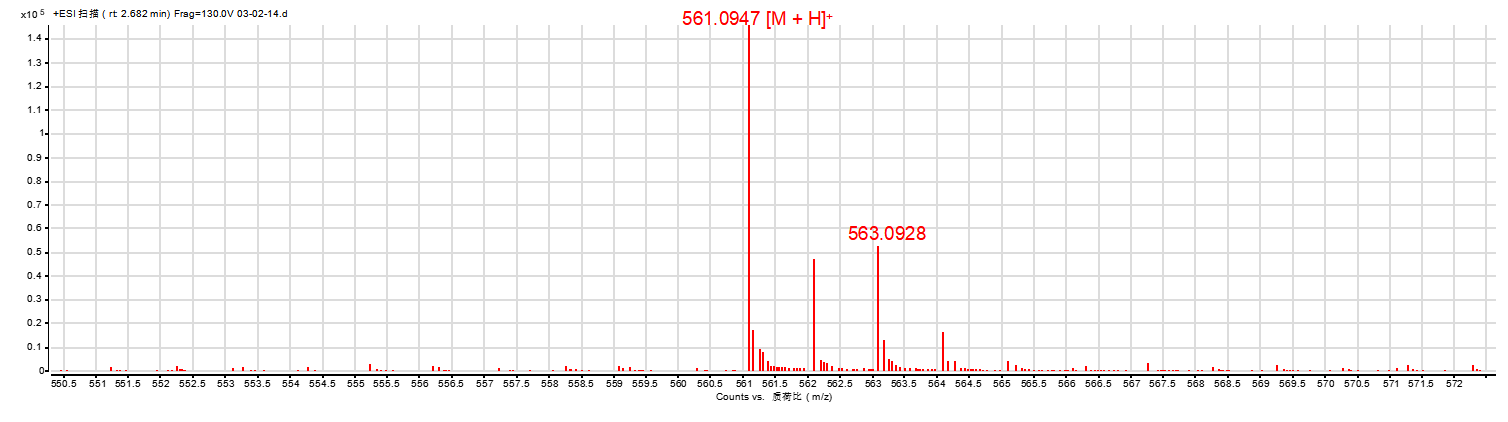
Figure S11.** HRESIMS spectrum of penithrone C (**3**).


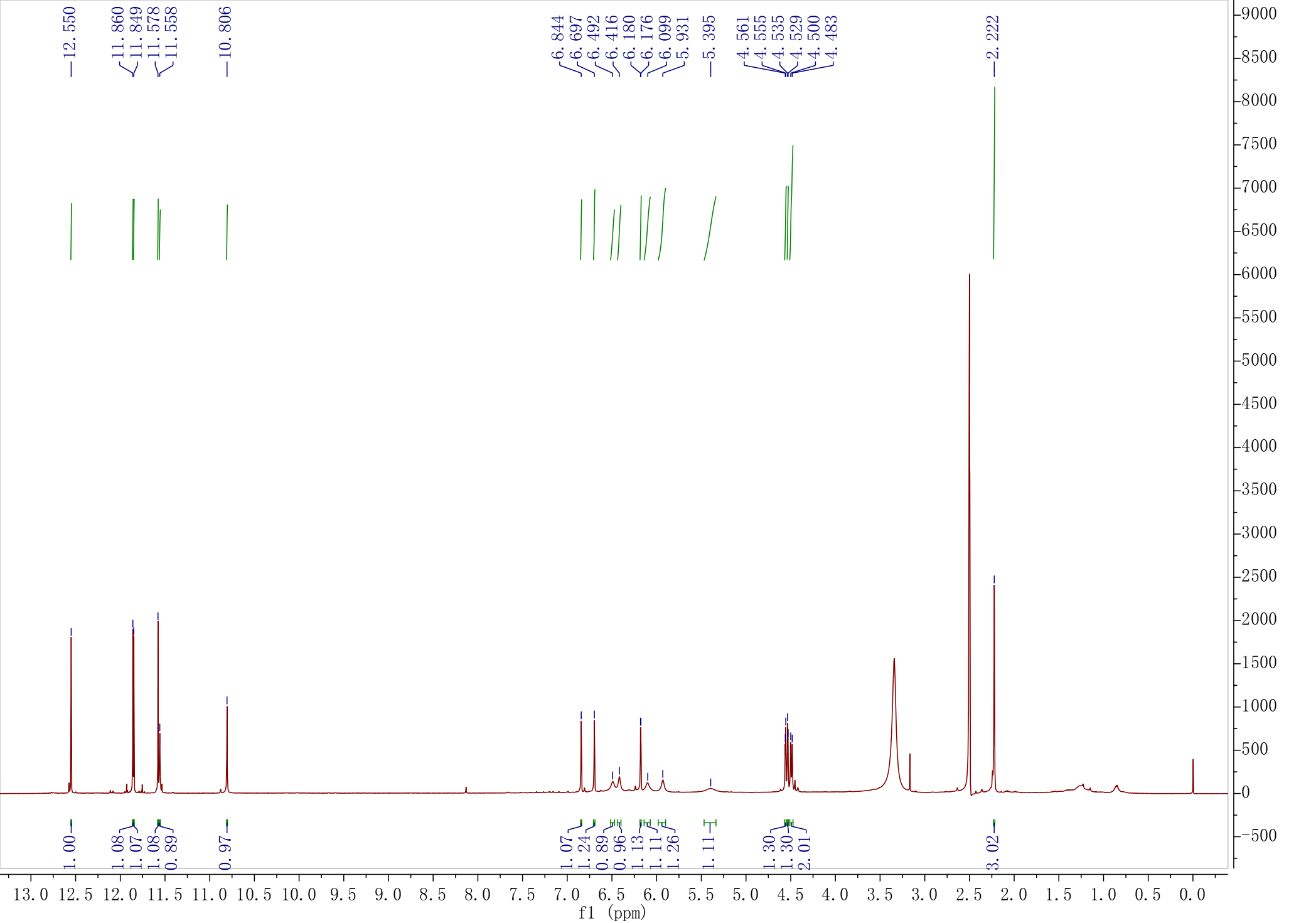


**Figure S12**. ^1^H NMR spectrum of penithrone C (**3**; 500 MHz, DMSO).


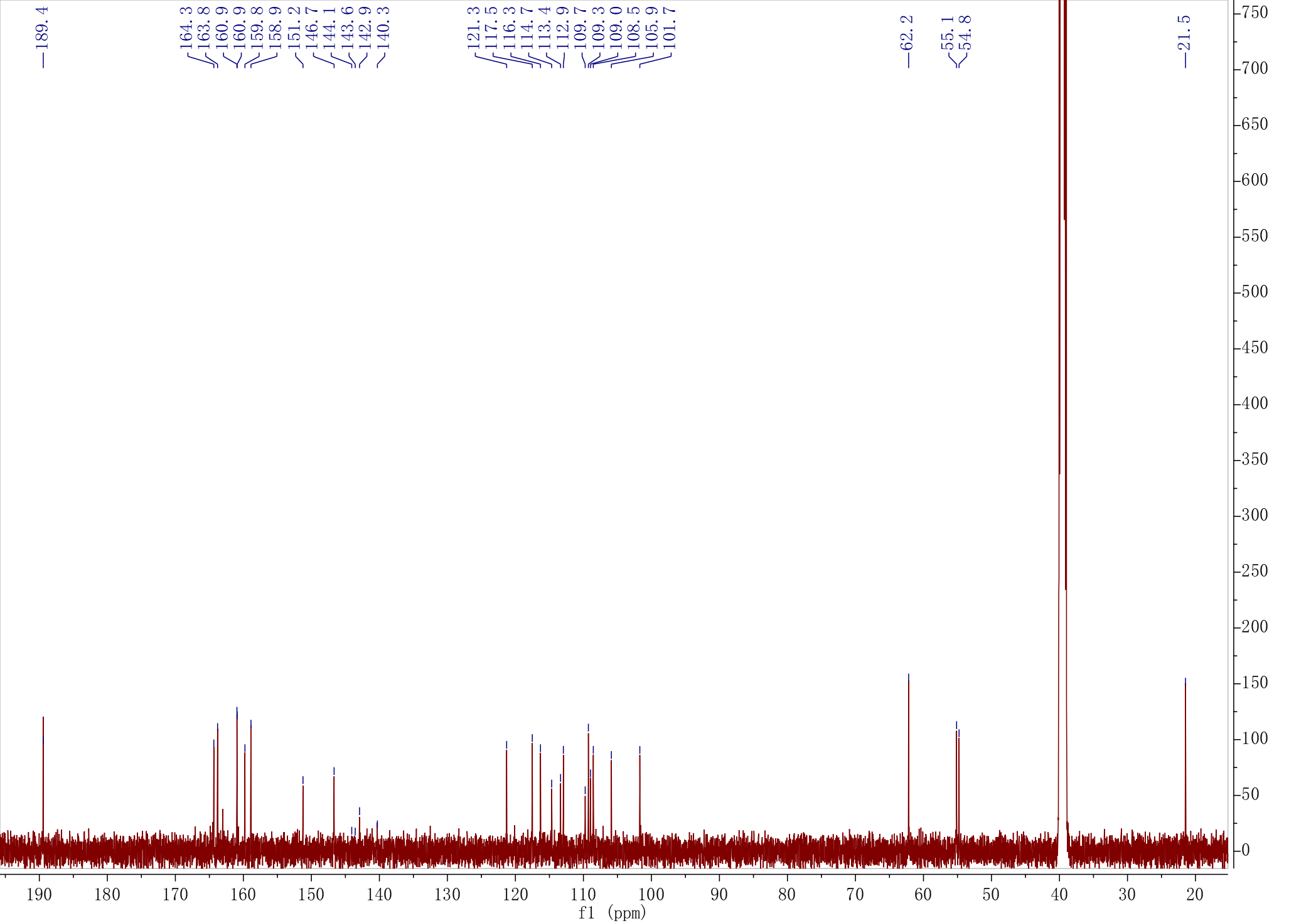


**Figure S13**. ^13^C NMR spectrum of penithrone C (**3**; 125 MHz, DMSO).


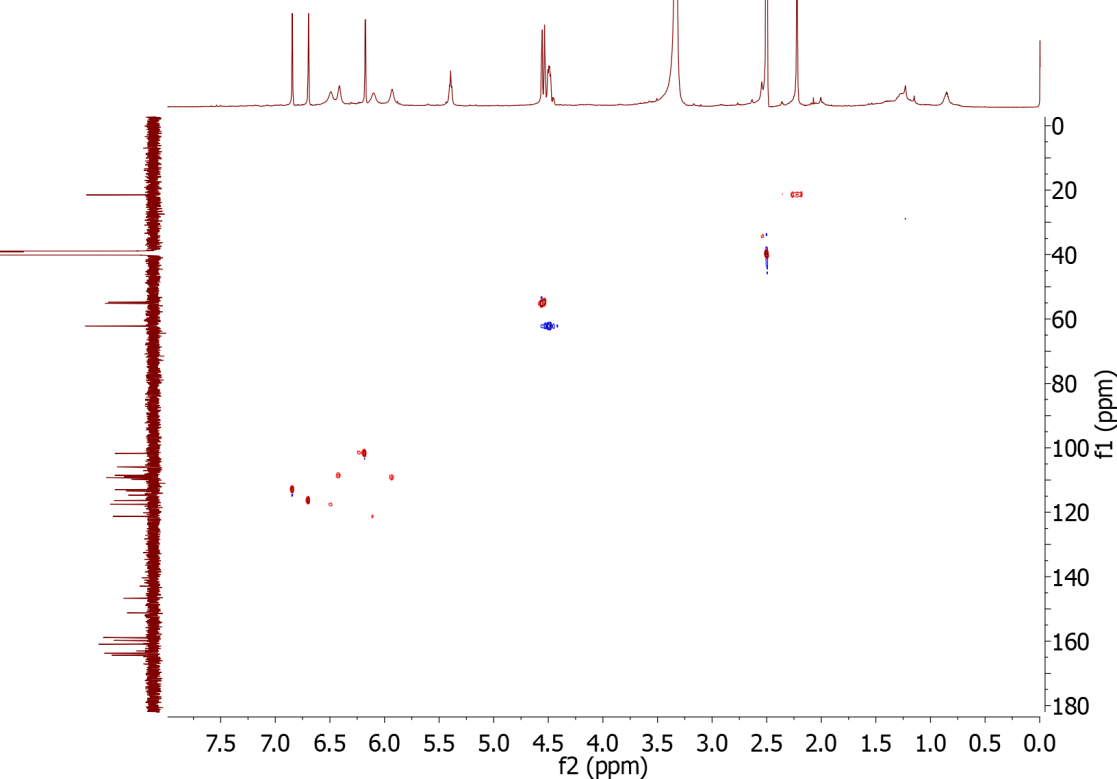


**Figure S14**. HSQC spectrum of penithrone C (**3**; 500 MHz, DMSO).


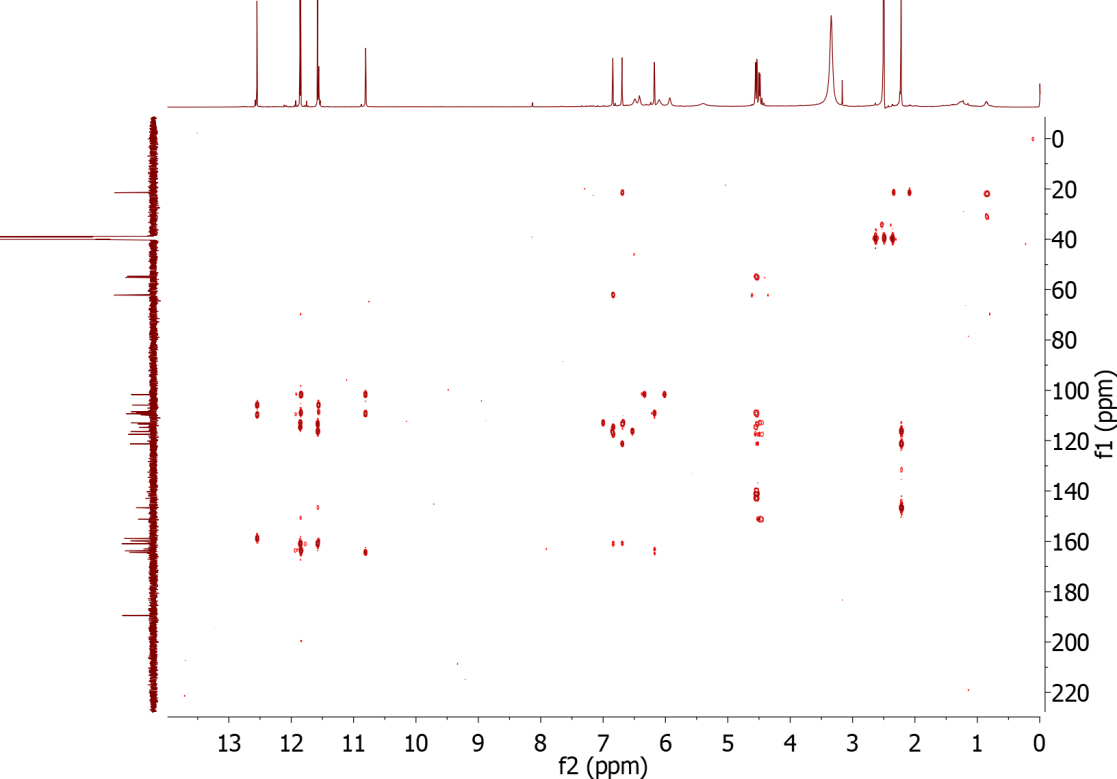


**Figure S15**. HMBC spectrum of penithrone C (**3**; 500 MHz, DMSO).

|   *syn* | |   *anti* | |
| --- | --- | --- | --- |
| Conformers | Populations (%) | Conformers | Populations (%) |
| 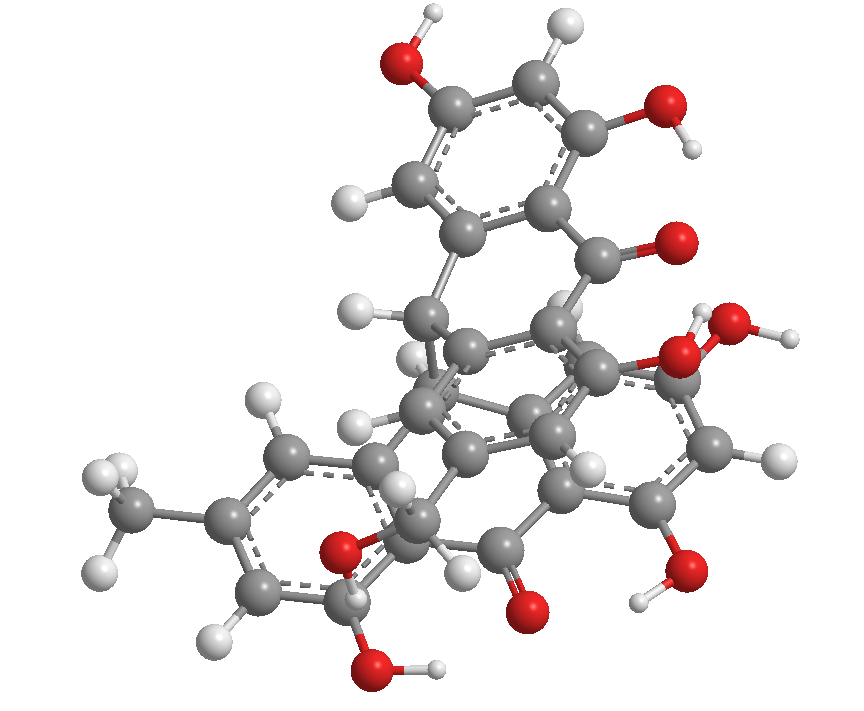 | 19.71 | 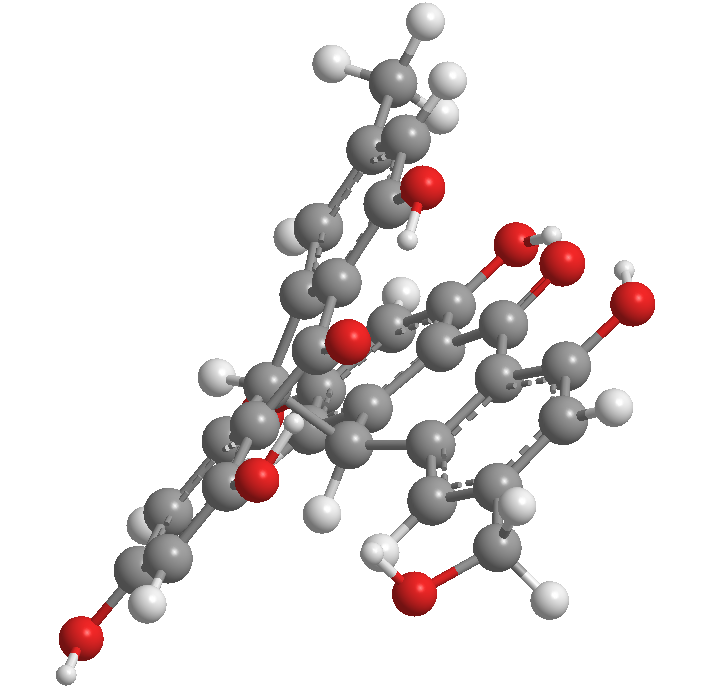 | 22.05 |
| 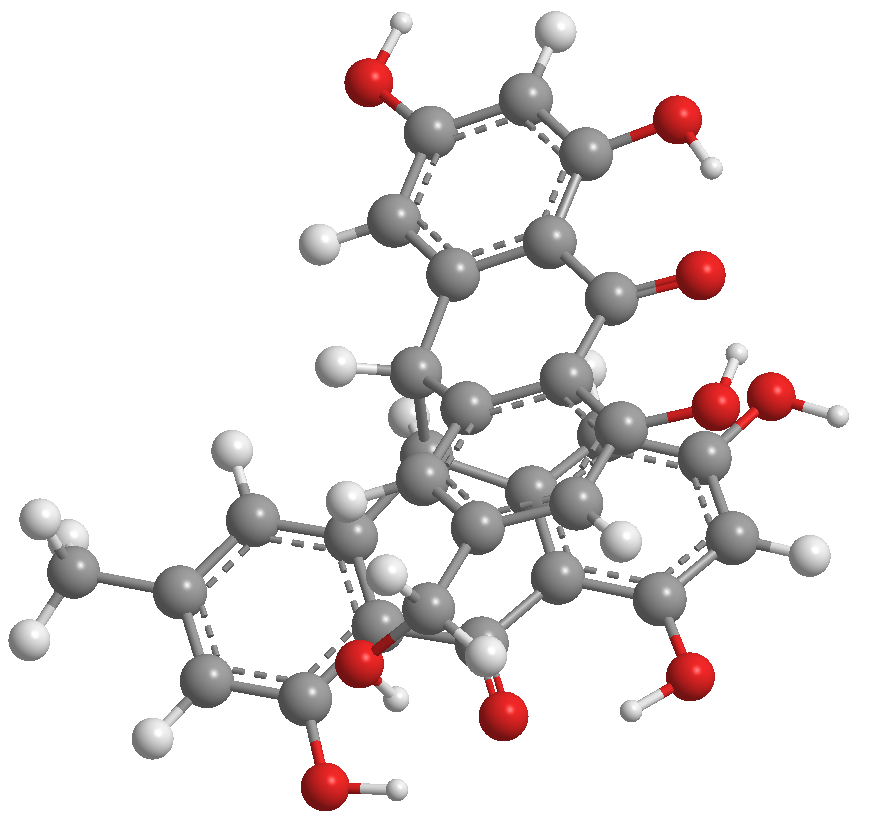 | 12.45 | 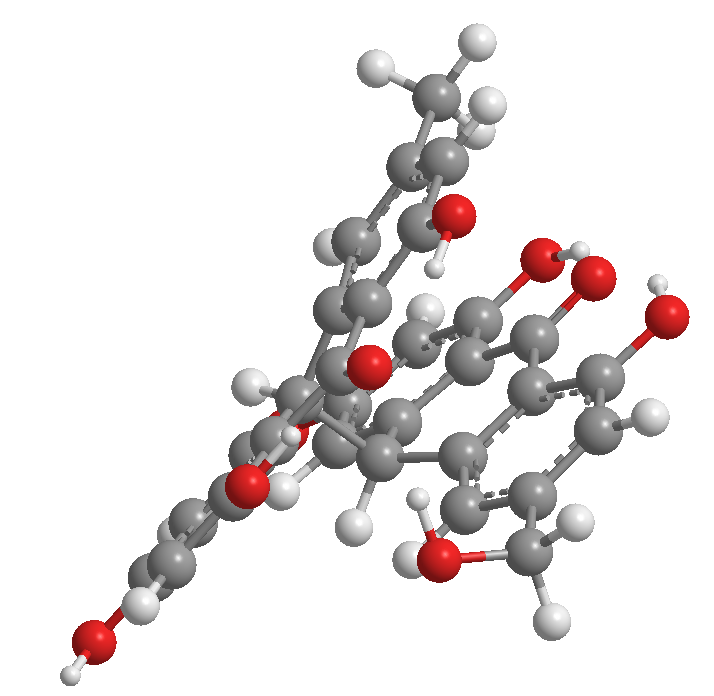 | 20.81 |
| 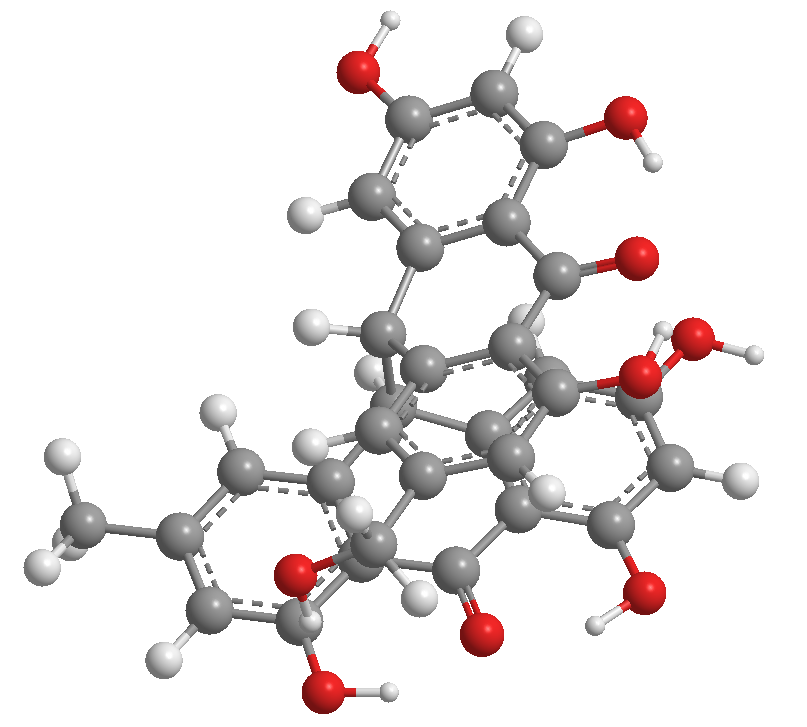 | 22.45 | 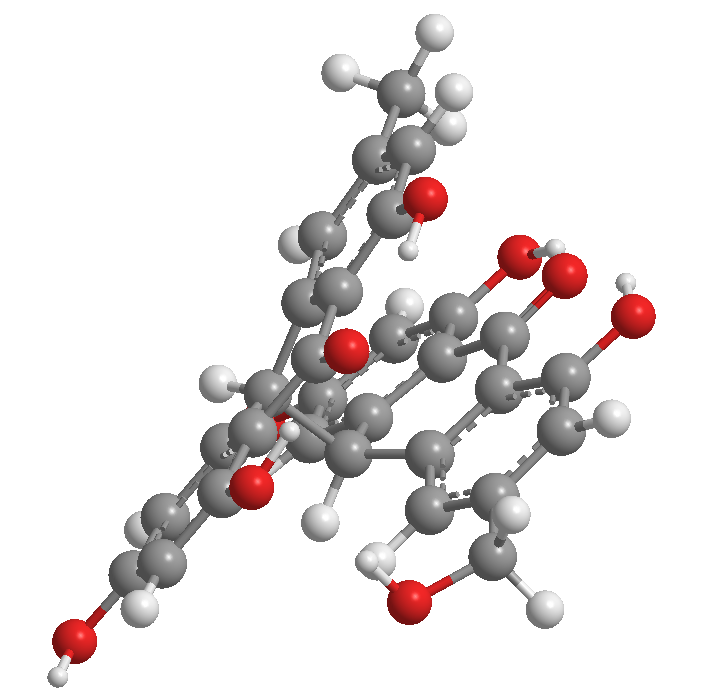 | 20.29 |
| 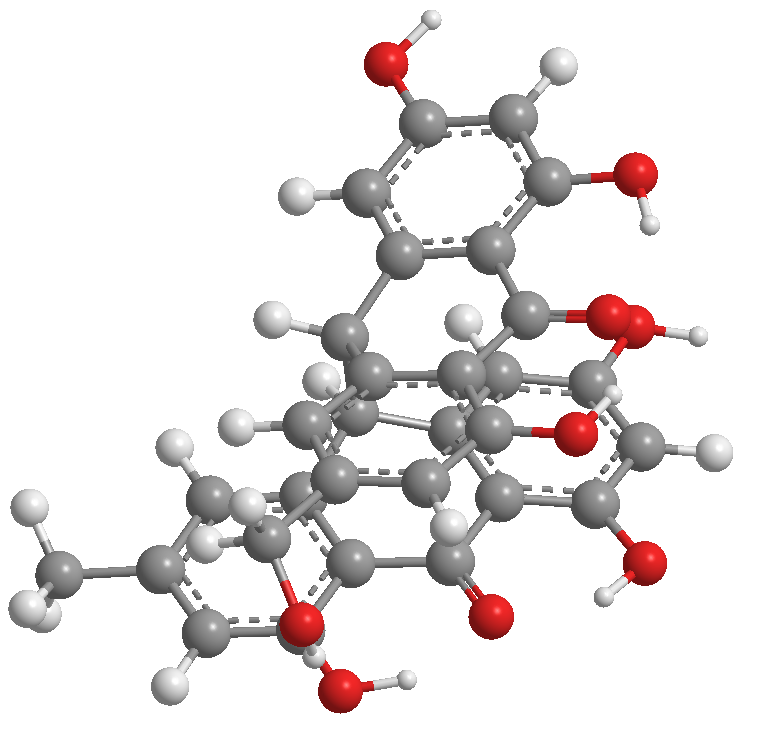 | 2.03 | 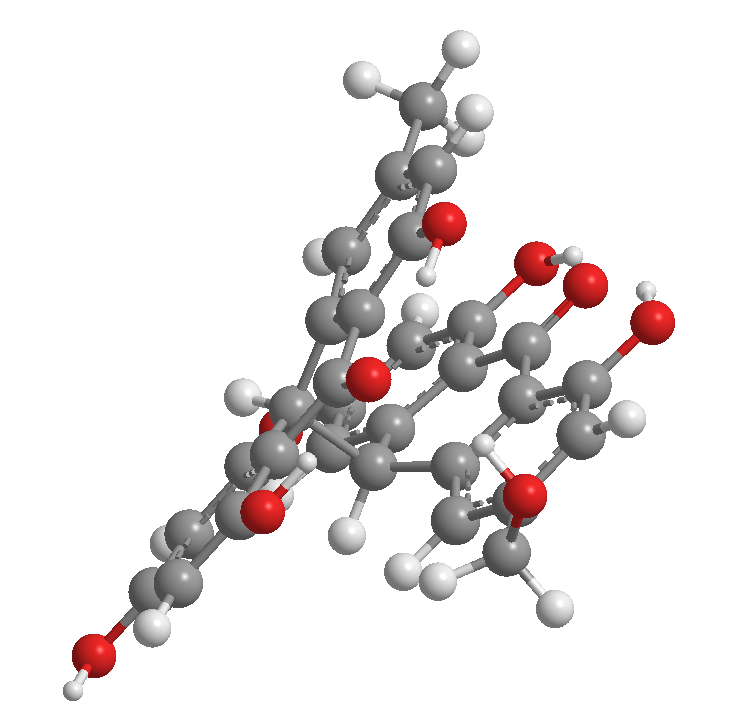 | 8.39 |
| 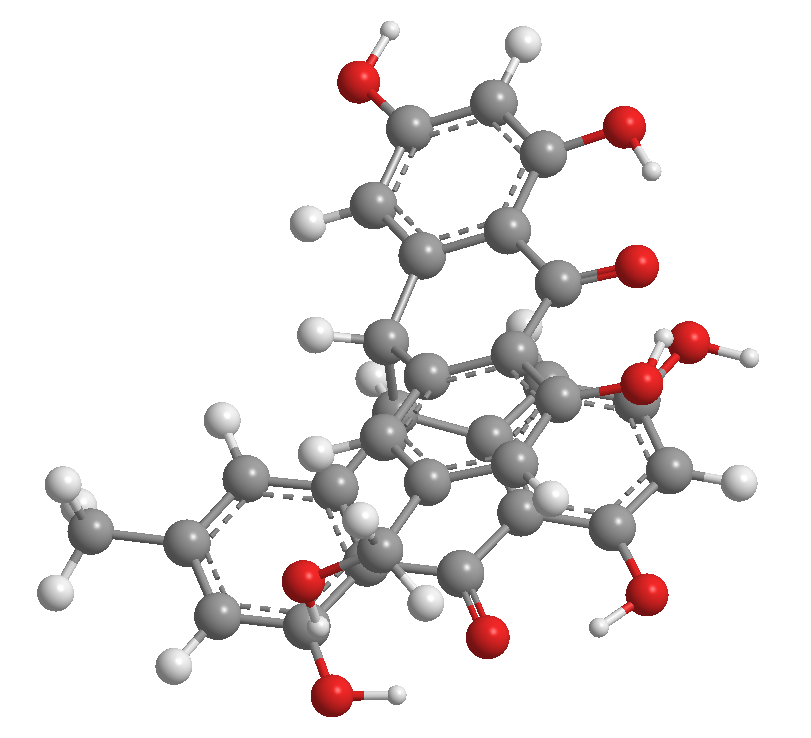 | 20.54 | 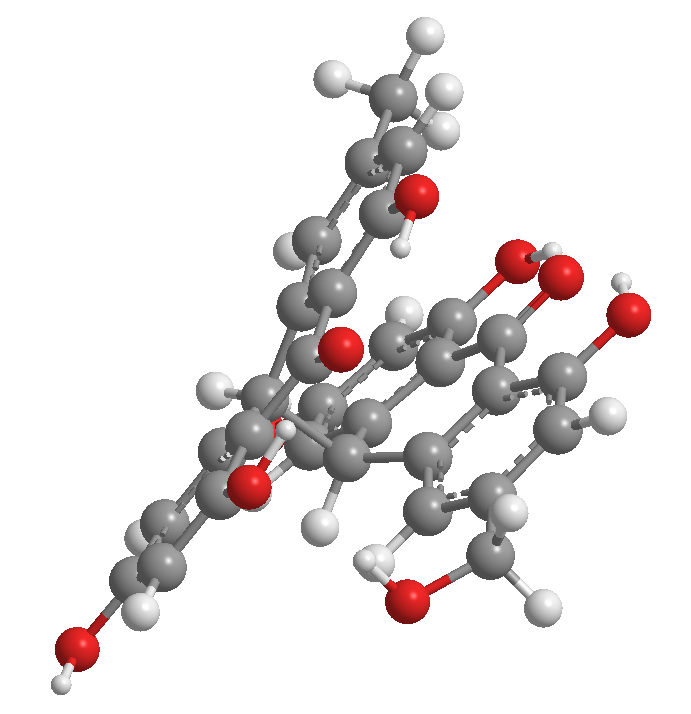 | 20.55 |
| 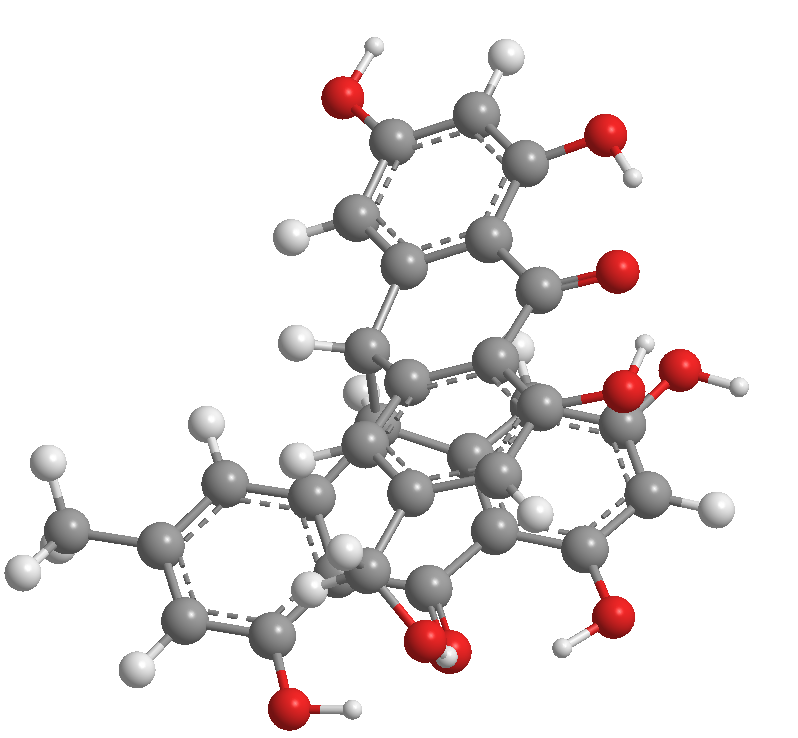 | 8.64 | 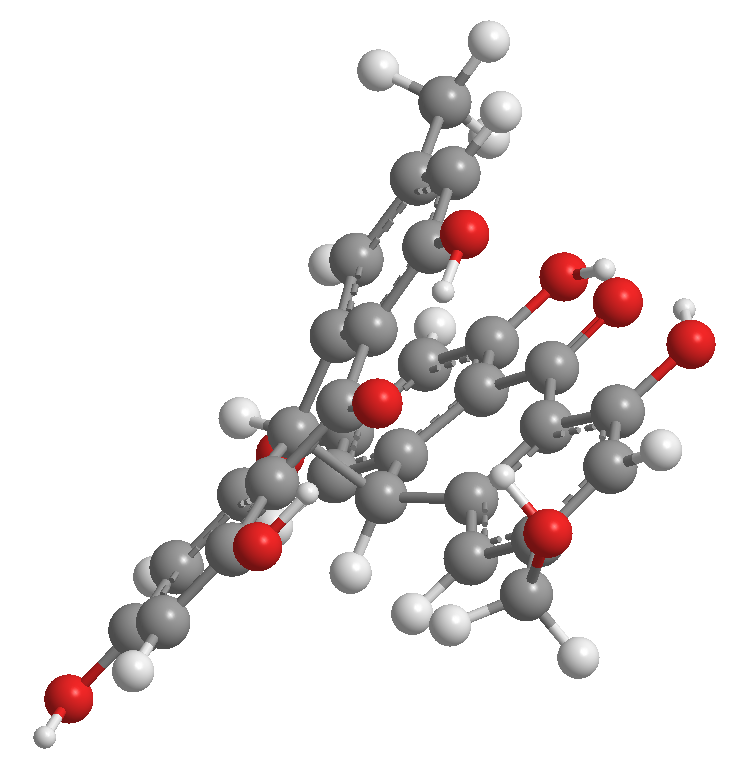 | 7.91 |
| 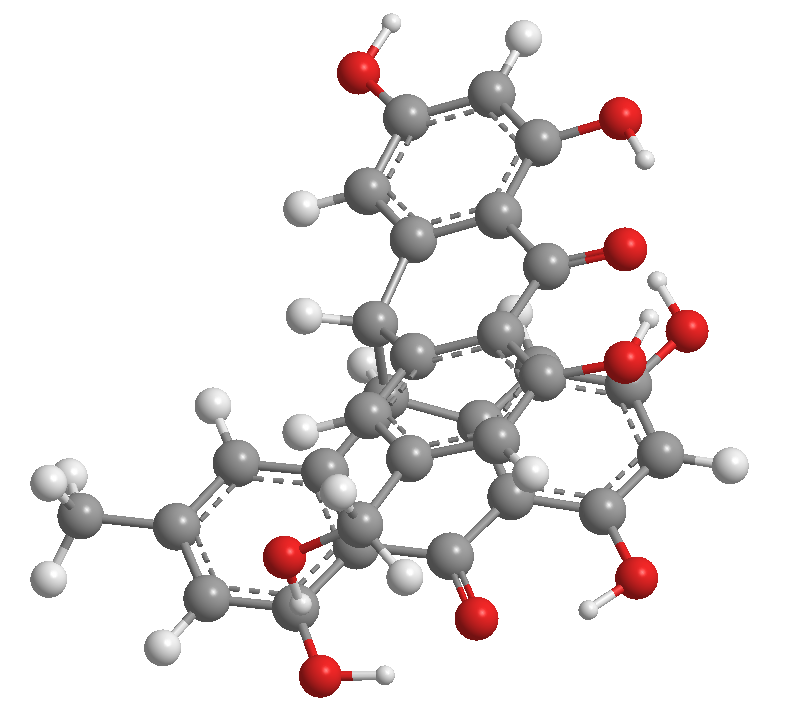 | 14.18 |  |  |

**Figure S16.** The optimized conformers in NMR calculation for *syn* isomer and *anti* isomer.
